# Regulation of ERK-MAPK signaling in human epidermis

**DOI:** 10.1186/s12918-015-0187-6

**Published:** 2015-07-25

**Authors:** Joseph Cursons, Jerry Gao, Daniel G. Hurley, Cristin G. Print, P. Rod Dunbar, Marc D. Jacobs, Edmund J. Crampin

**Affiliations:** Systems Biology Laboratory, Melbourne School of Engineering, University of Melbourne, Melbourne, Australia; NICTA Victoria Research Lab, Melbourne, Australia; ARC Centre of Excellence in Convergent Bio-Nano Science and Technology, Melbourne School of Engineering, University of Melbourne, Melbourne, Australia; Auckland Bioengineering Institute, University of Auckland, Auckland, New Zealand; Maurice Wilkins Centre, University of Auckland, Auckland, New Zealand; Bioinformatics Institute, University of Auckland, Auckland, New Zealand; Faculty of Medical and Health Sciences, University of Auckland, Auckland, New Zealand; School of Biological Sciences, University of Auckland, Auckland, New Zealand; Department of Biology, New Zealand International College, ACG New Zealand, Auckland, New Zealand; School of Mathematics and Statistics, University of Melbourne, Melbourne, Australia; School of Medicine, University of Melbourne, Melbourne, Australia

## Abstract

**Background:**

The skin is largely comprised of keratinocytes within the interfollicular epidermis. Over approximately two weeks these cells differentiate and traverse the thickness of the skin. The stage of differentiation is therefore reflected in the positions of cells within the tissue, providing a convenient axis along which to study the signaling events that occur *in situ* during keratinocyte terminal differentiation, over this extended two-week timescale. The canonical ERK-MAPK signaling cascade (Raf-1, MEK-1/2 and ERK-1/2) has been implicated in controlling diverse cellular behaviors, including proliferation and differentiation. While the molecular interactions involved in signal transduction through this cascade have been well characterized in cell culture experiments, our understanding of how this sequence of events unfolds to determine cell fate within a homeostatic tissue environment has not been fully characterized.

**Methods:**

We measured the abundance of total and phosphorylated ERK-MAPK signaling proteins within interfollicular keratinocytes in transverse cross-sections of human epidermis using immunofluorescence microscopy. To investigate these data we developed a mathematical model of the signaling cascade using a normalized-Hill differential equation formalism.

**Results:**

These data show coordinated variation in the abundance of phosphorylated ERK-MAPK components across the epidermis. Statistical analysis of these data shows that associations between phosphorylated ERK-MAPK components which correspond to canonical molecular interactions are dependent upon spatial position within the epidermis. The model demonstrates that the spatial profile of activation for ERK-MAPK signaling components across the epidermis may be maintained in a cell-autonomous fashion by an underlying spatial gradient in calcium signaling.

**Conclusions:**

Our data demonstrate an extended phospho-protein profile of ERK-MAPK signaling cascade components across the epidermis *in situ*, and statistical associations in these data indicate canonical ERK-MAPK interactions underlie this spatial profile of ERK-MAPK activation. Using mathematical modelling we have demonstrated that spatially varying calcium signaling components across the epidermis may be sufficient to maintain the spatial profile of ERK-MAPK signaling cascade components in a cell-autonomous manner. These findings may have significant implications for the wide range of cancer drugs which therapeutically target ERK-MAPK signaling components.

**Electronic supplementary material:**

The online version of this article (doi:10.1186/s12918-015-0187-6) contains supplementary material, which is available to authorized users.

## Background

**Fig. 1:**
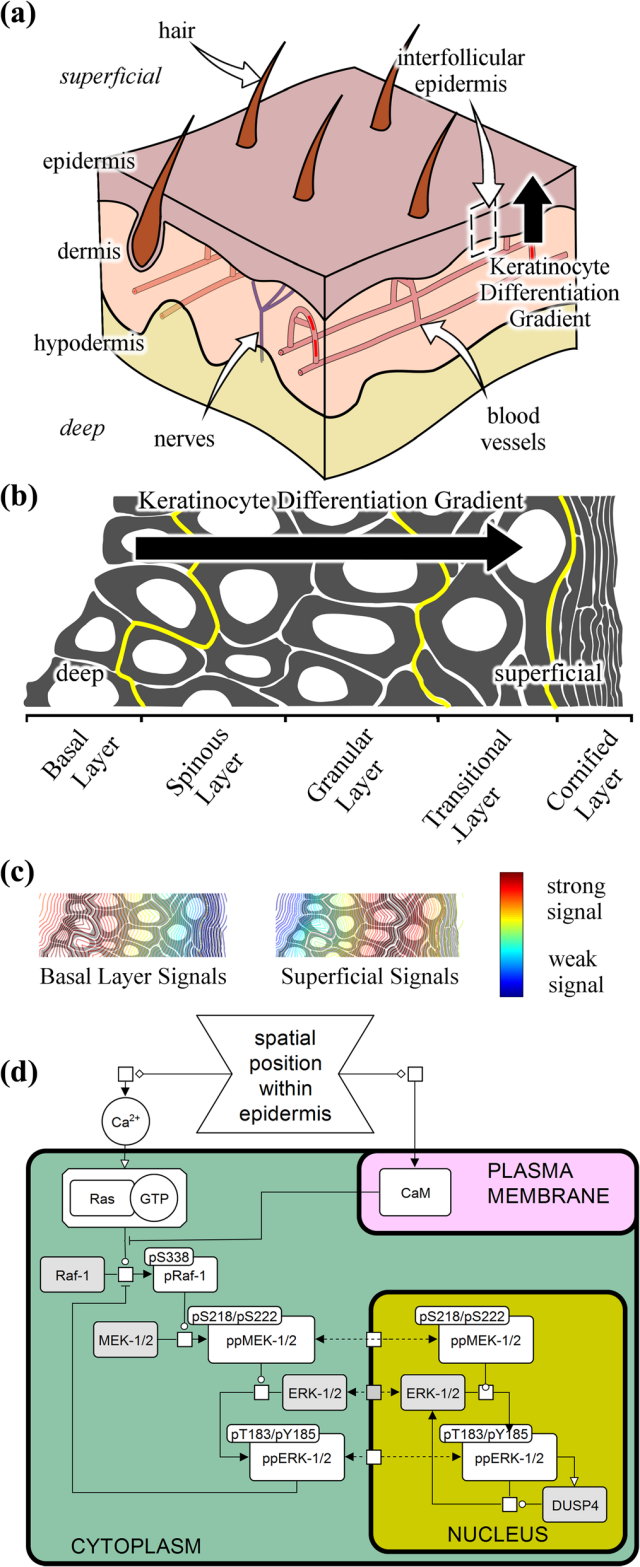
ERK-MAPK Signaling Within Human Epidermis. **a** Epidermis is the outermost tissue layer of the skin with an essential role in protection from the environment. Epidermal barrier function is established and maintained by keratinocytes which undergo large biochemical and morphological changes during keratinocyte terminal differentiation. This establishes a spatially-regulated keratinocyte differentiation gradient across the depth of the epidermis, between hair follicles (within interfollicular epidermis). **b** Differentiating keratinocytes are pushed towards the superficial surface of the epidermis by proliferation within the basal layer. As this occurs, keratinocytes undergo terminal differentiation, establishing a spatiotemporal differentiation gradient across the depth of the epidermis. **c** The effect of tissue structure on paracrine/endocrine signals, and differentiation-associated changes in the abundance or activity of scaffold co-factors establish signal gradients across the depth of the epidermis. The gradient of Ca^2+^ within the epidermis is similar to the ‘superficial signals’ example; however, it peaks just prior to the transitional layer, rather than within the outermost superficial layers. **d** A simple representation of the canonical ERK-MAPK signaling cascade with: inputs to Raf-1 from extracellular calcium (Ca^2+^; activating) and plasma membrane calmodulin (CaM; inhibiting) modulated by cellular position along the keratinocyte differentiation gradient; the signal transduction cascade through Raf-1, MEK-1/2 and ERK-1/2; negative feedback from phospho-ERK-1/2 to phospho-Raf-1; and nuclear phospho-ERK-1/2 promoting its own dephosphorylation. Further details on these interactions are given within the Materials and methods. Nodes drawn in *grey* are not explicitly modeled, as they were not measured experimentally. A more comprehensive reaction kinetic scheme is given within Additional file 7: Figure S1 in reaction_network.png

The epidermis is an epithelial tissue which forms the outermost layer of the skin (Fig. 1a) and performs an essential role in protecting an organism against environmental perturbations [1]. Interfollicular human epidermis consists of keratinocytes arranged in a spatial gradient of differentiation across the deep-to-superficial axis of the tissue (Fig. 1b). Epidermal barrier function is dependent upon changes to keratinocyte biochemistry and morphology that occur during differentiation [1, 2] and discrete tissue layers (Fig. 1b) are defined by histological features that reflect underlying molecular changes [1, 3–7]. A variety of signaling proteins are also modulated across the epidermis to coordinate keratinocyte terminal differentiation (Fig. 1c), including extracellular matrix ligands and associated plasma membrane receptors [8–10], and calcium (Ca^2+^) signaling components [11, 12]. Of particular interest for this study, increasing extracellular Ca^2+^ ion concentration is known to promote keratinocyte terminal differentiation *in vitro* [13, 14], and a Ca^2+^ gradient is maintained across the depth of the epidermis, increasing from the basal layer to the outermost granular layer (similar to ‘Superficial Signals’ in Fig. 1c) before decreasing across the transitional layer [11, 12].

The canonical extracellular signal regulated kinase (ERK) cascade of the mitogen activated protein kinase (MAPK) family has been implicated in the regulation of keratinocyte differentiation *in vivo* [15–17] and *in vitro* [18, 19]. Signaling through ERK-MAPK integrates and mediates the effects of epidermal growth factor receptor (EGFR) [19, 20], integrin [21, 22] and calcium signaling [18, 23]. These input stimuli are localized to the plasma membrane where they regulate the conversion of Ras-GDP to active Ras-GTP. Generation of Ras-GTP promotes signaling through a sequential cascade of kinases which are activated by phosphorylation prior to phosphorylating their own downstream targets, progressing through activation of Raf-1 and B-Raf dimers, to MEK-1/2 and then ERK-1/2 (Fig. 1d). Phosphorylated ERK-1/2 then act upon a large number of proteins including several transcription factors to activate gene expression and initiate a cellular response [16, 24]. The sub-cellular localization of activated ERK-MAPK components is also important for determining specific cellular responses to the wide range of signals that influence ERK-MAPK signaling [25, 26].

The ERK-MAPK cascade has been extensively characterized using *in vitro* models [27–30] and computational methods [25, 31–33], typically over a time course of minutes-to-hours following addition of a mitogenic stimulus [27] or some other perturbation [29]. A number of studies have modeled intracellular signaling during developmental pattern formation, primarily using *Drosophila* and other malleable systems [34, 35]. Within human tissues however, the temporal and spatial dynamics of ERK-MAPK signaling, and the role that it may play in regulating cellular processes to help maintain tissue homeostasis are not well understood. Keratinocytes take approximately two weeks to traverse the epidermis as they undergo terminal differentiation [36–38], raising the question as to how ERK-MAPK signaling operates over this much longer timescale *in situ*. Furthermore, cells grown *in vitro* are exposed to the different environmental cues than cells in their *in vivo* tissue, and this can have significant effects on phenotypic behavior and intracellular signaling. For example, epidermal keratinocytes grown *in vitro* undergo abnormal differentiation expressing proteins more commonly associated with wound-healing and basal cell carcinoma [39–41].

In this study we therefore determined to characterize ERK-MAPK signaling in human epidermis *in situ*, by measuring abundance of signaling pathway components and mathematically modeling the spatiotemporal dynamics of the signaling cascade. We used immunofluorescence to measure the abundance of phospho-Raf-1, phospho-MEK-1/2, phospho-ERK-1/2 and calmodulin (CaM) within healthy human skin samples. A keratinocyte’s position within the epidermis, along the deep-to-superficial axis of the tissue, directly reflects its relative stage of differentiation. We used this to develop a computational framework for transforming the image data into a quantitative format, which allowed us to combine observations across multiple experiments. Our results show gradual, coordinated increases in the abundance of ERK-MAPK phospho-proteins over the depth of the skin, over much longer timescales than those observed for *in vitro* studies. Statistical analyses highlighted the importance of considering spatial position within the tissue when examining relationships between the measured variables and suggested canonical interactions are active during keratinocyte differentiation. Thus, we constructed a mathematical model of ERK-MAPK signaling interactions derived from the literature, using a normalized-Hill differential equation approach [42, 43], with which to analyze our data. The purpose of this model was to test whether Ca^2+^ signaling components which vary over the depth of the epidermis were sufficient to drive signaling through canonical ERK-MAPK interactions and reach different steady-state activation levels, in a manner which was consistent with the extended spatiotemporal phosphorylation profiles observed within our data. Our results suggest that canonical ERK-MAPK interactions are operating, as overall pathway activity is modulated through the depth of the epidermis.

## Results

### Spatial transformation of immunofluorescence data using histological landmarks reveals distinct spatial profiles of in situ ERK-MAPK phosphorylation within human skin

**Fig. 2:**
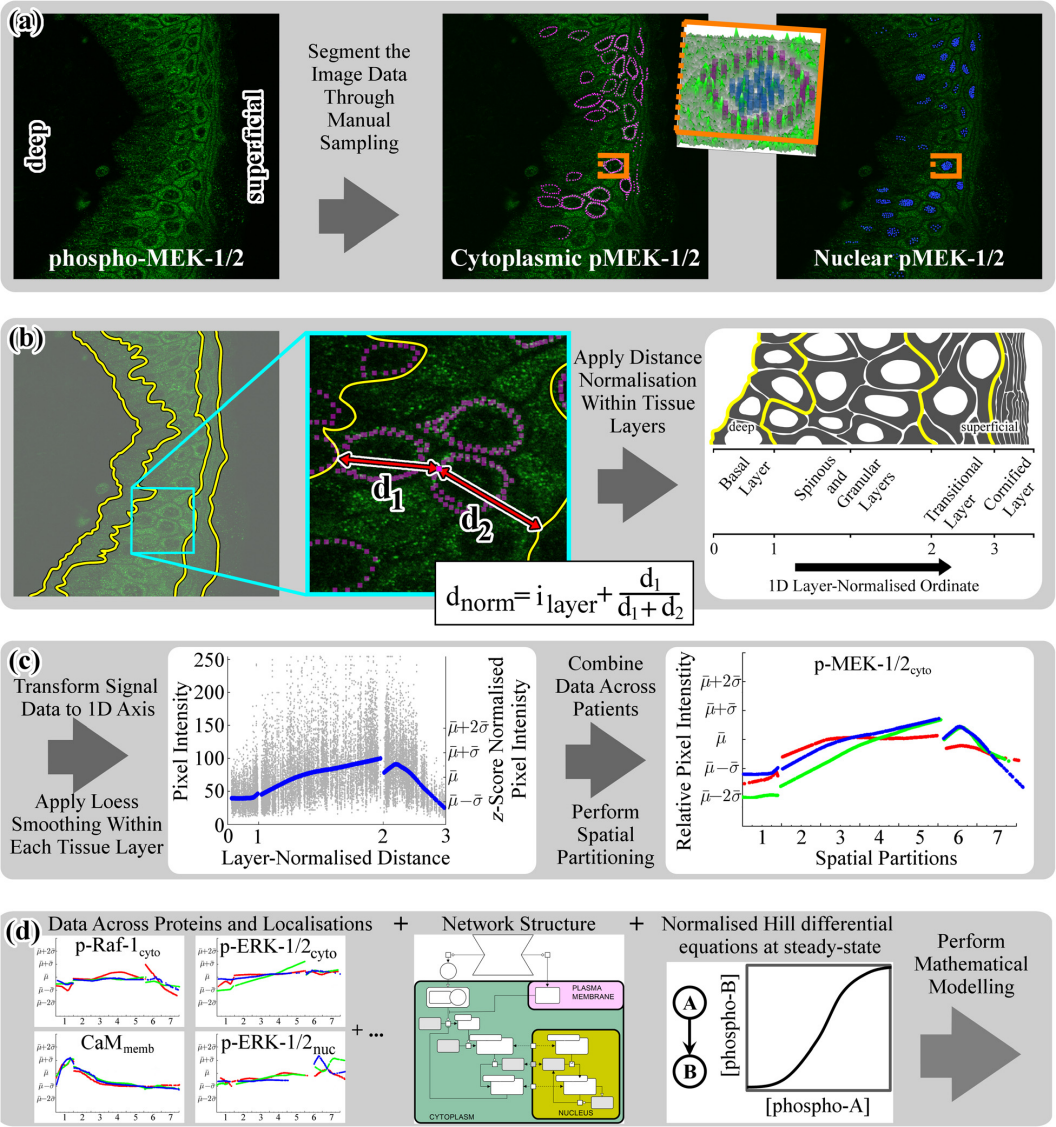
Transforming fluorescence image data in to a spatially-conditioned, quantitative format for mathematical analysis. **a** Image data were obtained by immunofluorescence labeling and confocal microscopy, and different sub-cellular localizations (cytoplasm and nucleus) were manually sampled using a graphical user interface. The position and orientation of the cell selected for surface rendering (*inset*) has been highlighted (*orange lines*). **b** Epidermal tissue layers that could be distinguished using label-independent criteria were demarcated. The relative position of each sample was normalized within the layer using linear interpolation, then added to a whole integer which distinguished tissue layers (i_layer_; Table 1), such that the normalized distance, d_norm_ = i_layer_ + (d_1_/(d_1_ + d_2_)). **c** The sampled fluorescence intensity data (*grey dots*) underwent loess smoothing (*blue line*). Spatial conditioning allowed data from Patients One (*red*), Two (*green*) and Three (*blue*) to be directly compared. **d** Protein abundance data from specific sub-cellular compartments were compared to a normalized-Hill differential equation model, with a literature derived network structure (described in Fig. 1d). This model was solved to steady-state at different spatial positions through the epidermis

 We used confocal microscopy imaging of immunofluorescence-labeled human epidermis to measure the abundance of ERK-MAPK phospho-proteins and calmodulin within interfollicular keratinocytes from samples of human skin (Additional file 1: Figure S2, Additional file 2: Figure S3, Additional file 3: Figure S4, Additional file 4: Figure S5 in pRaf1.png; pMEK.png; pERK.png; CALM.png). Regions of interest were manually selected within the image data using a GUI which displayed the images and samples at different *z*-positions and tracked input co-ordinates through ‘mouse clicks’. This provided a relatively quick approach to sample the image data across multiple patients and targets, while distinguishing distinct subcellular domains of the cytoplasm, nucleus (Fig. 2a), and where possible the plasma membrane (Additional file 4: Figure S5 in CALM.png).

Images of the epidermis were segmented into discrete tissue layers using histological features apparent with single-target labeling (Fig. 2b; yellow lines) as detailed in our previous work [44]. Next, we introduced a one dimensional ‘layer-normalized’ ordinate which followed the spatiotemporal gradient of keratinocyte terminal differentiation, while minimizing the effect of variations in tissue layer thickness (Fig. 2b) caused by rête ridges (epidermal extensions in to the underlying dermis) and inter-patient differences in the absolute thickness of the epidermis (Additional file 5: Figure S6 in PatALL_-_CompThickness.png). This layer-normalized distance mapped to whole integers at tissue layer boundaries (Table 1), and the relative position within a tissue layer for each sampled region was calculated by linear interpolation, using minimum distances to the deep (*d*_*1*_) and superficial (*d*_*2*_) boundaries of each tissue layer (Fig. 2b). The computational approach to transform fluorescence image data onto a layer-normalized ordinate made the data more comparable across patients (Fig. 2c) and target proteins (Fig. 2d). Fluorescence intensity data reflecting protein abundance were extracted from the sampled pixels and mapped to the layer-normalized distance prior to loess smoothing (Fig. 2c). Smoothed loess curves calculated from these data therefore reflect trends for protein abundance variation across the depth of the epidermis.

**Table 1:** Spatial partitioning scheme of epidermal tissue layers

	Layer-normalized distance		
	0–1	1–2	2–3
Total spatial partitions	Basal layer	Spinous and granular layers	Transitional layer
7	1	2–5	6 & 7
28	1–4	5–20	21–28

The layer-normalized distance was converted from a continuous measure along the gradient of keratinocyte terminal differentiation into a discrete number of ‘spatial partitions’ as illustrated in Fig. 2c. These spatial partitions better reflect the relative number of cells within each layer, with a ratio of 1:4:2; one basal cell, four spinous and granular cells and two transitional cells

To use these data for subsequent modeling (Fig. 2d), we mapped normalized signal intensity data from the layer-normalized distance on to discrete spatial partitions which capture the relative number of cells within each tissue layer (Fig. 2c, *x*-axes; Table 1; further details given in Materials and methods–Image data processing).

### Spatial profiles of ERK-MAPK signaling components indicate varying states of the signaling pathway in keratinocytes across the epidermis

**Fig. 3:**
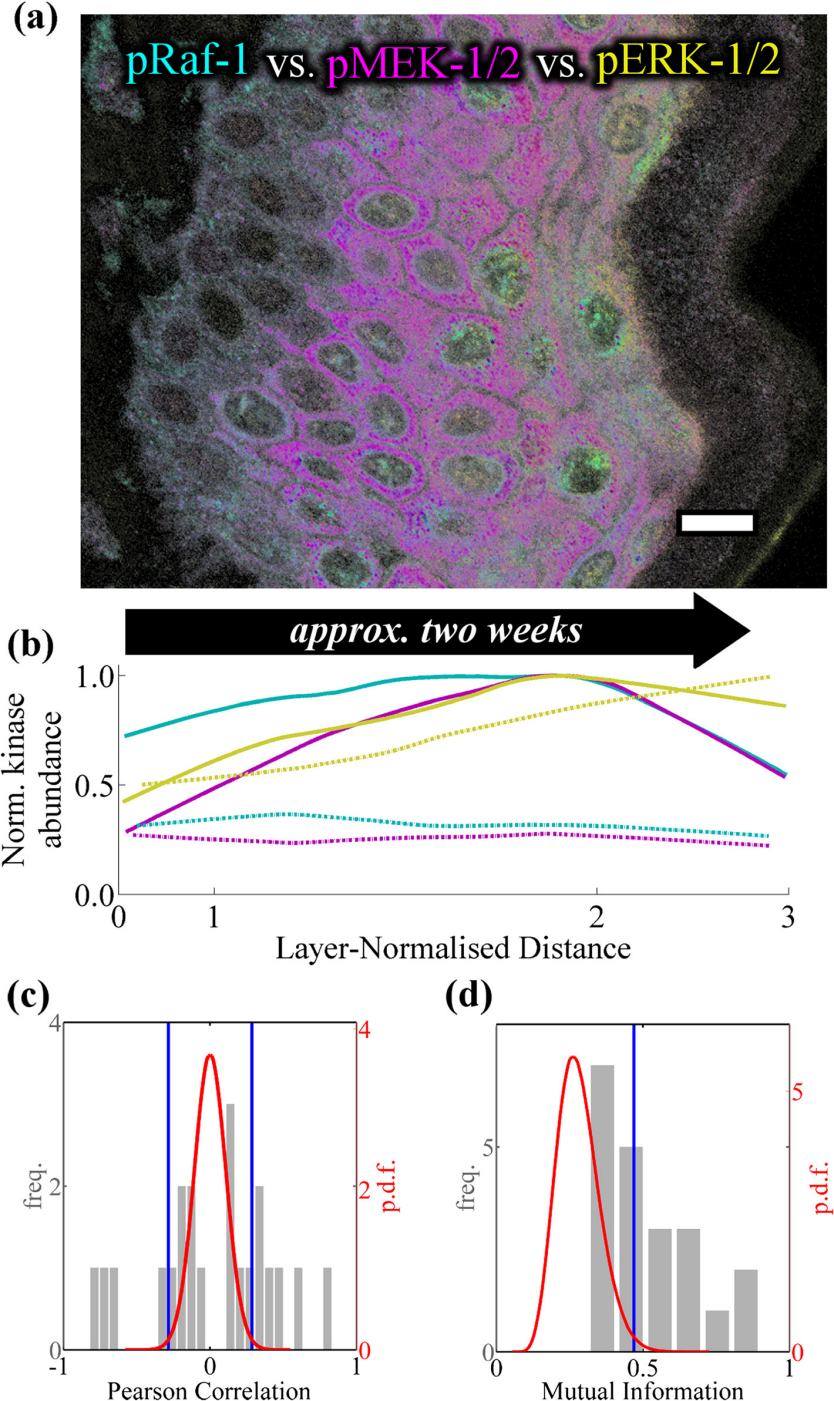
Spatially coordinated changes to phosphorylated ERK-MAPK components within human epidermis. **a** Human epidermis simultaneously labeled against phospho-Raf-1 (pS338; *cyan*), phospho-MEK-1/2 (pS218/pS222; *magenta*) and phospho-ERK-1/2 (pT183/pY185; *yellow*). *Scale bar* represents 10 μm, image data have undergone non-linear transformation to improve printed appearance. **b** The normalized abundance of cytoplasmic (*solid lines*) and nuclear (*dashed lines*) phospho- Raf-1, −MEK-1/2, and ERK-1/2 (*colors as above*) within interfollicular keratinocytes undergoing terminal differentiation *in situ*. The **c** Pearson’s correlation and **d** mutual information were calculated between all pairwise combinations of target variables using the spatially-conditioned abundance data (*grey histograms*; *axes at left*). The strength of these statistical associations was compared to a null distribution calculated from spatially-scrambled data (*red probability density function [p.d.f]*; *axes at right*), which was used to calculate two-sided (*Pearson’s correlation*) and one-sided (*mutual information*) 99 % confidence intervals (*blue vertical lines*). For details on the strength of individual relationships please refer to Additional file 6: Table S1

For *in vitro* studies the ERK-MAPK signaling cascade is usually activated by a mitogenic stimulus and changes in protein phosphorylation are examined over minutes-to-hours. Our immunofluorescence data suggest that phosphorylated ERK-MAPK components change in a coordinated manner over the depth of the epidermis (Fig. 3a & b), and thus over a much longer timescale than *in vitro* studies. The notion that cells within different parts of a tissue behave in a different yet coordinated manner is supported by qualitative observations throughout the literature, thus, we attempted to qualify our observation that ERK-MAPK is modulated across the depth of the epidermis in a quantitative manner.

Our image processing framework transforms fluorescence intensity data onto the layer-normalized distance ordinate (Fig. 2), producing ‘spatially-conditioned’ data, where the abundance of each target is described with respect to relative spatial position within the epidermis. To test the significance of this spatial conditioning, phospho-protein abundance data were randomly resampled along the spatial axis. These scrambled data were used to establish a null distribution describing the strength of statistical association which could be expected from data with the same marginal distributions (*i.e.* those arising by chance), in the absence of any spatial information.

We identified the pairwise relationships between phosphorylated ERK-MAPK components which have a spatially-conditioned statistical association which is greater than associations observed within the spatially-scrambled null distribution (Fig. 3c & d; *p*-value ≤0.01). Many are well-studied, canonical signaling interactions, suggesting that these relationships may be active over the gradient of keratinocyte terminal differentiation (Additional file 6: Table S1). This result highlights the importance of considering spatiotemporal information when examining intracellular signaling mechanisms *in situ*. Furthermore, given the two week time course associated with keratinocyte terminal differentiation [36–38], this result suggests that phosphorylation of the ERK-MAPK cascade is being regulated over this extended timescale *in situ*.

### Mathematical modeling of ERK-MAPK signaling across the epidermis is consistent with the canonical signaling cascade operating in a quasi-steady-state manner

To investigate whether canonical ERK-MAPK interactions identified *in vitro* can explain the extended phosphorylation gradient reported here, we use a normalized-Hill differential equation approach [43] to model ERK-MAPK signaling (Fig. 2d). This simplified modeling approach is largely dependent upon the network structure and directionality of interactions defined within the model, making it attractive for our system.

The signaling network (Fig. 1d & Additional file 7: Figure S1 in reaction_network.png) contained the canonical Raf-MEK-ERK interactions with MEK and ERK explicitly separated into cytoplasmic and nuclear compartments with shuttling. Tissue Ca^2+^ and plasma membrane CaM were included as an activator and inhibitor of cytoplasmic phospho-Raf-1, respectively [18, 23]. Feedback mechanisms within the ERK-MAPK cascade were also incorporated, namely an inhibitory feedback from cytoplasmic phospho-ERK-1/2 to cytoplasmic phospho-Raf-1 [45], and the self dephosphorylation of nuclear phospho-ERK-1/2 through inducing the expression of the nuclear localized ERK phosphatase DUSP4 [46]. State variables are defined as the fractional activation of each signaling species relative to an arbitrary maximal activity (*e.g.* for ‘ERK’ we examine phospho-ERK-1/2 activity levels).

**Fig. 4:**
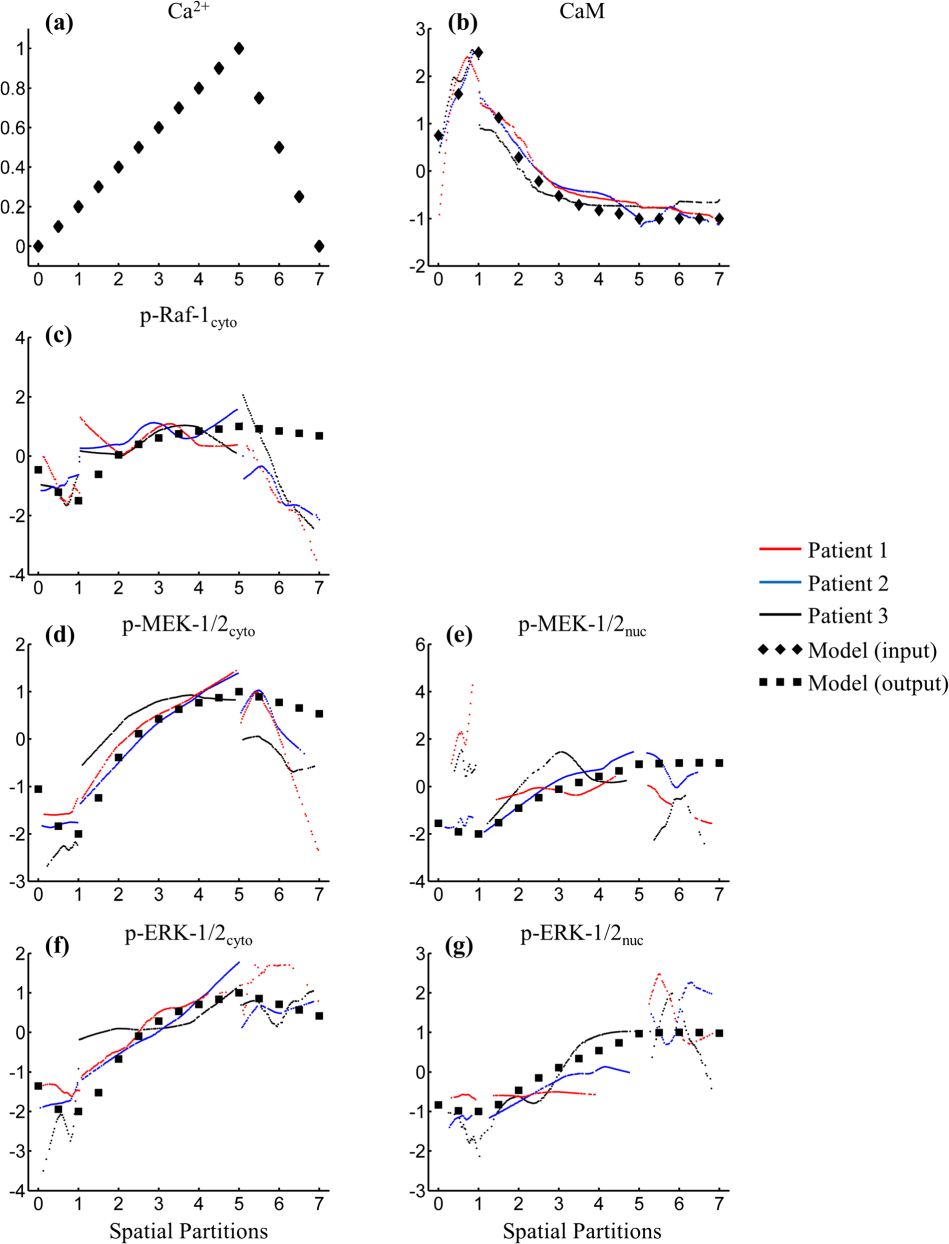
Simulated and measured abundance profiles of species in our ERK-MAPK signaling model across human epidermis. Our model of the ERK-MAPK pathway is stimulated by: **a** normalized epidermal Ca^2+^ as derived by Mauro *et al.* [11]; and **b** plasma membrane CaM abundance as derived from our experimental data (Additional file 4: Figure S5i in CALM.png). At each spatial position, the model was run to steady-state with these inputs and **c**-**g** the simulated relative abundance of ERK-MAPK components is compared to our *in situ* experimental measurements. The *y*-axis in (**a**) has normalized units, and where experimental data is plotted (**b**-**g**), the *y*-axis represents the *z*-score of the immunofluorescence pixel intensities. The model input and output abundances were manually scaled to visually fit the experimental data by adjusting the baseline level and amplitude

Relative calcium concentrations (Ca^2+^) through the depth of the epidermis (Fig. 4a) were derived from the literature [11], and the spatial profile of plasma membrane calmodulin (CaM) (Fig. 4b) was derived from our immunofluorescence data (Additional file 4: Figure S5 in CALM.png). The activity of these input species was used to stimulate the *in situ* fractional activation of Raf-MEK-ERK using normalized-Hill differential equations. Using spatial steps of ‘half-cell’ width across the epidermis (*i.e.* steps of 0.5 across 7 spatial partitions; Table 1) we generate conditions for the cell signaling cascade model at 15 spatial locations. For each of these 15 conditions, the model was then integrated until a steady-state was reached. This allows us to determine whether spatial variation in the modeled ‘inputs’ to the signaling cascade can maintain the observed spatial profile of ERK-MAPK activation across the epidermis. Only four parameters were optimized during the least-squares fitting of cytoplasmic and nuclear phospho-ERK-1/2 immunofluorescence data: the baseline levels and amplitudes of Ca^2+^ and plasma membrane CaM (Table 2).

**Table 2:** Optimized model parameters

Species	Baseline level (*b*)	Amplitude (*a*)
Tissue Ca^2+^ (Ca)	0.754	0.092
Plasma membrane CaM (CaM)	0.363	0.485

Baseline level (*b*) and amplitude (*a*) parameters for the Ca^2+^ and plasma-membrane CaM fractional activations were optimized to produce the closest least-squares fit to the cytoplasmic and nuclear phospho-ERK-1/2 immunofluorescence data (for all three patients)

Qualitatively, the simulated trends for Raf-1, MEK-1/2, and ERK-1/2 fractional activation (Fig. 4c-g) are similar to our spatially-conditioned immunofluorescence data. Within the basal layer (spatial partition 1) the simulations predict relatively low levels of phosphorylation, which is consistent with the immunofluorescence data except for nuclear phospho-MEK-1/2 from two patients (patients 1 and 3). Across the spinous and granular layers (spatial partitions 2–5), there was very good agreement between simulated and measured components, both showing consistent increases in the abundance of the phosphorylated species.

The model predictions performed poorly within the outermost transitional layers (spatial partitions 6 & 7). In particular, the immunofluorescence data showed large decreases in the abundance of cytoplasmic phospho-Raf-1 and both cytoplasmic and nuclear phospho-MEK-1/2, but the model simulated little or no decrease in the phosphorylated abundance of these species. This can probably be attributed to the degradation processes activated during the final stages of keratinocyte differentiation [1], which are not described within our model.

Agreement between our immunofluorescence data and model predictions over the spinous and granular layers suggests that the network structure of the canonical signaling cascade with the specified feedback loops is sufficient to reproduce key features of the extended spatial phosphorylation gradient of ERK-MAPK components observed in human skin. As differentiating keratinocytes progress through the depth of the epidermis and change their expression of signaling receptors and adhesion molecules, the activity of signaling pathways within the keratinocytes shifts, and settles to a new, local steady-state of activation. In our model, we represent tissue microenvironment changes through the relative abundances of tissue Ca^2+^ and plasma-membrane CaM, and at each position in the epidermis we solve our simple model of the ERK-MAPK cascade within keratinocytes to steady-state. Modulation of ERK-MAPK phosphorylation over these extended timescales may play a central role in the pattern formation processes which establish and maintain epidermal homeostasis.

## Discussion

In this study, we developed a novel computational approach to quantify ERK-MAPK activation within human epidermis. Our layer-normalized ordinate incorporates histological landmarks which separate distinct epidermal tissue layers. This allowed us to combine protein abundances measured using single-target labeling into an aggregate data set for mathematical modeling. Previous studies have used the relationship between a keratinocyte’s position within the epidermis and its stage of differentiation, examining protein abundance data (fluorescence signal intensity) with respect to the position within the epidermis, and constructing ‘quantitative spatial profiles’ [47, 48]. Immunohistochemistry has also been applied to measure protein abundances within the skin and to quantify variation across different epidermal layers [49]. This is an important distinction between our work where multiple pattern formation processes maintain complex structures and intracellular signaling states, against studies of *in vitro* cell signaling where there is only random heterogeneity across the spatial domain.

For cell culture experiments a high degree of intercellular heterogeneity has been noted for ERK signaling, such that the population mean can provide a poor representation of the signal within individual cells [50]. Our statistical analysis (Fig. 3) shows that spatially-dependent changes along the gradient of cellular differentiation account for a large degree of the covariance in the phosphorylation of ERK-MAPK components (*i.e.* there is a relatively high mutual information between the abundance of successive phosphorylated pathway components). The regulation of signaling component compartmentalization is also thought to be critical for specificity of intracellular signaling pathway responses to different stimuli [26, 51], thus we distinguish the nucleus and cytoplasm when measuring the abundance of active ERK-MAPK components. We believe that methodologies using tissue-specific landmarks with information on subcellular localization, as described here, may be useful to combine data sets and investigate the role of intracellular signaling in the physiological functions of tissues.

Motivated by our statistical analysis, we constructed a model of ERK-MAPK interactions using normalized-Hill differential equations [42, 43]. During model fitting the equations for pathway interactions (*e.g.* phosphorylation) used default parameters, and only four parameters were optimized: the baseline levels and amplitudes of fractional activation for the input Ca^2+^ and CaM (Table 2). Given that many of our parameters were poorly constrained, and our signaling network contained several double-phosphorylation steps which can contribute to a non-linear response, the ability of the normalized-Hill approach to qualitatively reproduce *in situ* immunofluorescence data without extensive parameter-fitting was attractive. A primary limitation of this approach, however, is that it restricts our ability to interpret the modeling results in relation to the mechanistic rate constants of kinetic models. Furthermore, our simplified modeling approach could not examine the intercellular heterogeneity present within the data (Additional file 1: Figure S2, Additional file 2: Figure S3, Additional file 3: Figure S4, Additional file 4: Figure S5 in pRaf1.png; pMEK.png; pERK.png; CALM.png), as discussed in detail below.

The model we developed suggests that the canonical ERK-MAPK signaling cascade operating in a cell-autonomous manner (*i.e.* without direct cell-cell interactions or long range diffusion of ERK signaling molecules) can reproduce general features of the immunofluorescence data measured *in situ*, when driven by underlying changes in the surrounding tissue microenvironment. In particular, the relative abundances of tissue Ca^2+^ and plasma-membrane CaM were implemented as drivers of the model with spatial gradients, suggesting these molecules may be sufficient to establish the extended ERK-MAPK phosphorylation gradient we observed within homeostatic human epidermis. This is largely consistent with observations that increasing extracellular Ca^2+^ ion concentration promotes keratinocyte terminal differentiation *in vitro* [13]. It is further supported by the plasma-membrane localization of calmodulin within basal keratinocytes, where it may be down-regulating EGFR and Ras signaling to establish a threshold that prevents proliferation at low doses of growth factors [18]. When CaM-mediated inhibition of Raf was removed from the model, there was an increase in ERK-MAPK pathway activation, and the spatial profile of activation was much more linear, likely reflecting the input Ca^2+^ gradient (Additional file 8: Figure S7 in SEDML_EpidermalMAPK_varySpatPos_execTimeCourse_noCamRafInhib-output_ScatPlot.png).

Although calcium signaling alone appeared sufficient to drive the observed gradient of ERK-MAPK activation in our model, we acknowledge that this does not contain all possible mechanisms which may influence ERK-MAPK signaling or keratinocyte differentiation. A wide array of signals regulate keratinocyte terminal differentiation [8–12] and mathematical methods have been used to identify dysregulated feedback mechanisms which contribute to a variety of skin pathologies [52–54]. Our model does not attempt to explain the mechanisms that establish and maintain the epidermal Ca^2+^ gradient [12, 14], in part, because increasingly complex mechanisms are still being identified, such as circadian-rhythm regulated changes in transcript abundance which influence the response of keratinocyte stem cells to Ca^2+^signaling [55]. Furthermore, due to the ‘single time point’ nature of our *in situ* image data we also cannot consider intracellular Ca^2+^ oscillations with epidermal keratinocytes [56, 57].

The model provides a good match to the data across the spinous and granular layers where the gradient of keratinocyte differentiation is more gradual and continuous (Fig. 4c-g). Failure to match the data within the basal layer may be partially attributable to heterogeneity in Ca^2+^ abundance [12] imposed by asynchronous oscillations, such that small fractions of cells are undergoing different cell processes at any given time-point. Differences in short timescale processes such as this oscillatory behavior may have contributed to variation in the basal layer signaling states that were observed for Patients One and Three (Fig. 4). Rule-based formalisms have given insight towards TGF-β1 regulation of keratinocyte proliferation during wound healing [58] and have been used to test alternative hypotheses for the regulation of basal keratinocyte proliferation [59]. Coupling multiple agent-based models together with tissue-gradient data may provide an approach to further examine the tissue-level effects of intercellular heterogeneity and shorter timescale processes such as the Ca^2+^ oscillations described above.

An aim of this study was to investigate how the ERK-MAPK cascade extensively studied *in vitro* may contribute to the maintenance of tissue structure in human epidermis. The pattern of ERK-MAPK signaling along the gradient of keratinocyte terminal differentiation appears to be largely consistent with epidermal biology and the ‘divergent effects’ of ERK-1/2 phosphorylation in regulating cell behavior. For example, transient activation of the ERK-MAPK cascade, typically observed *in vitro* after a relatively large dose of growth factors or other stimuli, tends to promote mitosis and cellular proliferation [18]. Within the basal layer of the epidermis, we observed relatively low ERK-MAPK activity, while a small number of cells show relatively high phospho-MEK abundance with nuclear localized signal (Additional file 2: Figure S3 in pMEK.png [red arrowheads] & Additional file 9: Figure S8 in FullCellSeg_pMEK_IntDistPDFs_edit.png). Unfortunately, due to the single-target labeling applied for this study, the relative activity of phospho-ERK within these cells cannot be directly examined. Furthermore, due to the relatively low frequency of phospho-MEK bright basal keratinocytes, we have not been able to identify such cells within the simultaneously-labeled tissue samples. Despite this, our observation that the basal epidermis contains a small number of phospho-MEK bright cells is consistent with the notion that basal keratinocytes are undergoing asynchronous division [10], such that at any one time, a small number of cells will show a transient increase in MEK activation just prior to G_2_/M progression [29]. This small number of proliferative basal keratinocytes with a relatively high nuclear phospho-MEK-1/2 abundance may have contributed to poor agreement between simulated and measured abundances (Fig. 4e).

As keratinocytes detach from the underlying basement membrane and begin execution of their terminal differentiation program, there is a small stepwise increase in the abundance of phospho-MEK-1/2 followed by a sustained, gradual increase over the spinous and granular tissue layers. Increased phosphorylation of ERK-1/2 has been observed with terminal differentiation in various cell lines [18, 60] and sustained ERK-MAPK pathway activation has been reported to induce growth arrest in epidermoid carcinoma cells [19]. It is tempting to speculate that sustained activation of the ERK-MAPK pathway helps to suppress cellular proliferation and ensure growth arrest in suprabasal keratinocytes by reducing the availability of unphosphorylated MEK-1/2 and ERK-1/2, preventing the transient increases that promote mitosis. The presence of complex regulatory processes described above, and the abnormal differentiation programs adopted by keratinocytes *in vitro *[39–41], make it difficult to test model predictions directly; however, transgenic mice with modified signaling components under inducible expression within the epidermis may be useful for testing such mechanisms [17, 61]. We believe that the observations in this work highlight the need for further studies of *in situ* signaling pathway activation.

## Conclusions

This study developed a novel, joint experimental-computational framework for investigating the regulation of ERK-MAPK signaling in homeostatic human epidermis. We have identified an extended phospho-protein gradient of ERK-MAPK signaling cascade components over the depth of the epidermis, and we utilized statistical analysis and mathematical modeling to demonstrate that canonical ERK-MAPK interactions driven by Ca^2+^ signaling components operating at quasi-steady-state are sufficient to establish this extended phosphorylation gradient. These findings may have implications for the wide range of cancer drugs which therapeutically target ERK-MAPK signaling components.

## Materials and methods

### Ethics statement

Human skin samples were obtained with written informed consent under a protocol approved by the New Zealand Northern Regional X Ethics Committee (project number NTX/08/09/086) and Counties-Manukau District Health Board (project number 681).

### Tissue collection

Fresh skin samples obtained from healthy patients undergoing plastic or reconstructive surgery were snap frozen in liquid nitrogen and stored at −80 °C.

### Immunofluorescence labeling and confocal microscopy

A detailed description of the immunofluorescence labeling and confocal microscopy protocols, including antibody catalogue numbers, are given elsewhere [44]. The antibody against calmodulin (UniProt: P62158) targets multiple isoforms. A phospho-serine 338 specific antibody was used to examine the activation state of Raf-1 (UniProt: P04049), as this phospho-epitope is routinely used as a surrogate marker for Raf-1 activation [45]. Antibodies against double-phosphorylated MEK-1/2 (pS218/pS222) and double-phosphorylated ERK-1/2 (pT183/pY185) were used to examine the abundance of active MEK-1/2 (UniProt: Q02750/P36507) and ERK-1/2 (UniProt: P27361/P28482).

Human epidermis was sectioned within a cryostat at −20 °C and immediately fixed with paraformaldehyde at room temperature, then labeled by indirect immunofluorescence and imaged using a Leica TCS SP2 confocal microscope. Secondary antibody control slides were imaged at the same gain and offset settings to ensure that the effects of non-specific labeling or auto-fluorescence were minimal. An imaging resolution at or exceeding the Nyquist sampling criteria was used, allowing the plasma-membrane, nucleus and cytoplasm of individual cells to be identified. Gain and offset values were adjusted to minimize underflow and overflow, and under these imaging conditions, fluorescence signal intensity was interpreted as an approximately linear measure of protein abundance within each voxel [62]. Image data were stored in an uncompressed .TIFF format.

### Image data processing

A collection of scripts for quantifying the image data was written using MATLAB (MathWorks, Natick MA). A graphical user interface was developed using the MATLAB image processing toolbox and the ginput2 function (MATLAB Central File Exchange FileID: #20645) to facilitate selection of regions of interest (ROIs). Tissue layer boundaries were specified using the path tool within GIMP (v. 2.4.7) and exported as a binarized .TIFF format. The minimum distance between ROIs and surrounding tissue layer boundaries was determined using the intrinsic MATLAB function dist. For analyses where data were combined across patients (Fig. 3), the signal intensity data were *z*-score normalized for each patient and then combined.

Loess smoothing was chosen due to its ability to cope with edge effects (by selecting local sample points dependent upon the smoothing frequency parameter) [63]. Loess smoothing was applied to the signal intensity data across each tissue layer using the intrinsic MATLAB function smooth. Although the normalized distance co-ordinate system captured the distinct epidermal layers well, signal intensity data examined against this gradient did not capture the different thicknesses of each tissue layer (Additional file 5: Figure S6 in PatALL_-_CompThickness.png). Thus, the spatial domain was further partitioned for discrete spatial sampling, in a manner that reflected the ratio of 1:4:2 cells between the tissue layers (Table 1).

### Statistical test of spatial conditioning

Image data (Fig. 3; Additional file 1: Figure S2, Additional file 2: Figure S3, Additional file 3: Figure S4) suggested that activation of the ERK-MAPK cascade occurs in a coordinated manner over the spatiotemporal gradient of keratinocyte terminal differentiation. To test this in a quantitative manner, a null hypothesis was proposed:

“the strength of statistical association between measured fluorescence signals (signaling component abundance) is independent of spatial conditioning”

Signal intensity data were extracted over 28 spatial partitions (Table 1). Data were examined over the spatial dimension, and the Pearson’s correlation and mutual information between each signaling component was calculated using the MATLAB corr and mutualinfo (MATLAB Central File Exchange FileID: #14888) functions, respectively. Data scrambling was applied to abrogate spatial conditioning of each variable while retaining the same marginal distributions of signal intensity data. For each target, signal intensity data were scrambled along the spatial domain (*i.e.* random sampling without replacement) 100,000 times. From these scrambled data, the Pearson’s correlation and mutual information were calculated between targets and the probability density functions were taken as null distributions. The two-sided (Pearson’s correlation) and one-sided (mutual information) 99 % confidence intervals were identified as thresholds (Fig. 3c & d; blue vertical lines). For the spatially-conditioned data, relationships with a statistical association exceeding these thresholds (Additional file 6: Table S1) were considered as evidence against the null hypothesis that the statistical associations are independent of spatial conditioning (*p* ≤ 0.01).

### Estimating cytoplasmic-to-nuclear volume ratio

Scripts were written using MATLAB to manually input sample points that demarcate the edge of cellular domains at various *z*-positions through the image data. A series of image processing functions were applied to: connect these data points and fill the selected area, smooth edges and subtract the nucleus from the cytoplasm, and interpolate the segmented areas between *z*-positions to produce a volumetric segmentation. From these data, the volume ratio of cytoplasm-to-nucleus was calculated for a number of cells across the depth of the epidermis, as shown in Additional file 10: Figure S10 in cyto_nuc_ratios_edit.png.

### Mathematical modeling

An SBML [64] implementation of the model has been deposited in BioModels Database [65] and assigned the identifier MODEL1503270000. Scripts used in this project, including SED-ML [66] to execute the model at different spatial positions and reproduce results from this paper are available from http://sourceforge.net/projects/EpidermalERKMAPK/.

The reaction kinetic diagram describing the signaling processes (Additional file 7: Figure S1 in reaction_network.png), and the simplified version of the network used for the model (Fig. 1d) were drawn as SBGN [67] process diagrams using VANTED [68]. To prevent over-parameterization of the model, indirect signaling processes that involve unmeasured components were ‘collapsed’ into a single arc. For example: Ca^2+^ is a direct input to phospho-Raf-1 within the model, as intermediate variables such as Ras-GTP were not measured; and similarly the role of nuclear phospho-ERK-1/2 in promoting its own dephosphorylation is modeled as a self-interaction, as the induced phosphatase DUSP4 was not measured.

Three types of interactions between species were used to construct the mathematical model:

1. *Activation*: Represented with a normalized activating Hill function [43]:
  1.1 $$ {f}_{act}(X)=\frac{B{X}^n}{K^n+{X}^n} $$
  where B and K are constrained such that f_act_(0) = 0, f_act_(EC_50_) = 0.5 and f_act_(1) = 1. EC_50_ is the fractional activation of an input species at which a half-maximal activation of an output species is induced;
2. *Inhibition*: Represented as the negative of *activation*;
3. *Dephosphorylation*: Represented as the product of *inhibition* with the fractional activation of the output species, as the abundance of the output species determines the maximal level of dephosphorylation that can occur.

In addition, shuttling between the cytoplasmic and nuclear compartments was modeled to be linearly proportional to the fractional activations of the species in the respective compartments. Pathway crosstalk was implemented by summing individual species interactions. Using this approach, the ERK-MAPK signaling network in Fig. 1d was represented with the following differential equations:

1.2 $$ \begin{array}{c}\hfill \frac{dRafc}{dt}=\frac{1}{\tau_{Rafc}}\left( \max \left({w}_{Ca, Rafc}{f}_{act}(Ca)-{w}_{CaM, Rafc}{f}_{act}(CaMm)-{w}_{ERKc, Rafc}{f}_{act}(ERKc),0\right)Raf{c}_{\max }- Rafc\right)\hfill \\ {}\hfill \frac{dMEKc}{dt}=\frac{1}{\tau_{MEKc}}\left({w}_{Rafc, MEKc}{f}_{act}(Rafc)MEK{c}_{\max }- MEKc-{s}_{MEKc n} MEKc+\frac{s_{MEKn c}}{r} MEKn\right)\hfill \\ {}\hfill \frac{dMEKn}{dt}=\frac{1}{\tau_{MEKn}}\left(- MEKn-{s}_{MEKn c} MEKn+r{s}_{MEKc n} MEKc\right)\hfill \\ {}\hfill \frac{dERKc}{dt}=\frac{1}{\tau_{ERKc}}\left({w}_{MEKc, ERKc}{f}_{act}(MEKc)ERK{c}_{\max }- ERKc-{s}_{ERKc n} ERKc+\frac{s_{ERKn c}}{r} ERKn\right)\hfill \\ {}\hfill \frac{dERKn}{dt}=\begin{array}{c}\hfill \frac{1}{\tau_{ERKn}}\Big(\left({w}_{MEKn, ERKn}{f}_{act}(MEKn)-{w}_{ERKn, ERKn}{f}_{act}(ERKn) ERKn\right)ERK{n}_{\max }- ERKn\hfill \\ {}\hfill -{s}_{ERKn c} ERKn+r{s}_{ERKc n} ERKc\Big)\hfill \end{array}\hfill \end{array} $$

where: state variables Ca, CaM, Rafc, MEKc, MEKn, ERKc and ERKn represent fractional activation of tissue Ca^2+^, plasma membrane CaM, cytoplasmic phospho-Raf-1, cytoplasmic phospho-MEK-1/2, nuclear phospho-MEK-1/2, cytoplasmic phospho-ERK-1/2, and nuclear phospho-ERK-1/2, respectively (note only active/phosphorylated ERK-MAPK components were examined in this study–although immunofluorescence data were collected for the non-phosphorylated forms, technical issues for MEK in particular prevented their inclusion within the quantitative modeling, as illustrated in Additional file 11: Figure S9 in MEK.png); *τ* is the time constant for a given species; *w* is the reaction weight; *X*_max_ is the maximal fractional activation of species; *s*_Xcn_ and *s*_Xnc_ are the cytoplasmic-nuclear and nuclear-cytoplasmic shuttling parameters for species *X*, respectively; and *r* is the cytoplasmic-nuclear volume ratio. In the equation governing cytoplasmic phospho-Raf-1 activity, the max function was incorporated to enforce that the inhibitory effects of plasma membrane CaM and cytoplasmic phospho-ERK-1/2 do not escalate into dephosphorylation effects instead (*i.e.* these inhibitions can only negate the activating effect of extracellular Ca^2+^, but cannot reduce fractional activation of cytoplasmic phospho-Raf-1). The scaling by r of the shuttling terms was to account for volume effects between the cytoplasm and nucleus. The cytoplasmic-nuclear volume ratio was set to vary linearly from 2 (deep) to 5 (superficial), as derived from the immunofluorescence data (Additional file 10: Figure S10 in cyto_nuc_ratios_edit.png).

Default parameters were used for the normalized Hill differential equations (n = 1.4, EC_50_ = 0.5, *τ* = 1, w = 1 and X_max_ = 1) [43]. MEK and ERK shuttling parameters (s_MEKcn_ = 0.05, s_MEKnc_ = 0.5, s_ERKcn_ = 0.01 and s_ERKnc_ = 0.01) were selected to reflect experimentally-determined ratios of cytoplasm-to-nucleus and nucleus-to-cytoplasm transport coefficients [27]. It is difficult to directly reconcile the parameter values derived for reaction kinetic models of individual cells undergoing rapid transient changes, and those used here for examining quasi-steady state changes over an extended timescale. One would expect similar shuttling rates associated with biochemical features, such as the nuclear export sequence on MEK [69]. There may be differences, however, as the HeLa cells used by Fujioka *et al.* [27] are derived from an adenocarcinoma and thus likely express different levels of scaffolding molecules/co-factors which vary between tissues [25, 70]. These issues highlight the need for studies which examine these signaling pathways within normal cells and homeostatic tissues.

The cell-autonomous signaling model (above) is driven by spatial profiles of extracellular Ca^2+^ and plasma membrane CaM activation levels across the epidermis. The Ca^2+^profile was modeled based on a previous experimental report [11] and CaM based on our own immunofluorescence data respectively as:

1.3 $$ \begin{array}{c}\hfill Ca={b}_{Ca}+{a}_{Ca}\left\{\begin{array}{ccc}\hfill d/5\hfill & \hfill for\hfill & \hfill d\in \left[0,5\right)\hfill \\ {}\hfill \left(7-d\right)/2\hfill & \hfill for\hfill & \hfill d\in \left[5,7\right]\hfill \end{array}\right.\hfill \\ {}\hfill CaMm={b}_{CaM}+{a}_{CaM}\left\{\begin{array}{ccc}\hfill \left(d+1\right)/2\hfill & \hfill for\hfill & \hfill d\in \left[0,1\right)\hfill \\ {}\hfill {e}^{\left(1-d\right)}\hfill & \hfill for\hfill & \hfill d\in \left[1,5\right)\hfill \\ {}\hfill 0\hfill & \hfill for\hfill & \hfill d\in \left[5,7\right]\hfill \end{array}\right.\hfill \end{array} $$

where: *b* and *a* are the baseline levels and amplitudes of the input fractional activations respectively (Table 2); *d* specifies the spatial partition (*i.e.* depth) within the epidermis.

A least-squares fit to the aggregate cytoplasmic and nuclear phospho-ERK-1/2 immunofluorescence data was performed with the MATLAB lsqnonlin and polyfit functions to optimize values of *b* and *a*. Differential equations were implemented in MATLAB (v7.12; R2011b) and solved numerically for each spatial partition using the ode23 function to find the steady-state (50 time units, with a time step of 0.1 time units).

## Additional files

Additional file 1: Figure S2. Human epidermis (Patient Two) labeled against phospho-Raf-1 (pS338) using immunofluorescence labeling with confocal microscopy imaging. (a, b) Immunofluorescence images are displayed together with (c) a surface rendering of the signal intensity within a suprabasal keratinocyte. The *z*-score normalized sampled signal intensity data (data points; plotted relative to the sample mean, $\overline{\mu}$; and sample standard deviation, $\overline{\sigma}$) and loess smoothed signals (solid lines) associated with the (d) cytoplasm and (f) nuclei are displayed for Patient One (red), Two (green) and Three (blue), together with the 90 % confidence interval for positive and negative residuals (dashed lines), and the sampled data clouds for Patient Two. Histograms of the discretized signal intensity within the (e) cytoplasm and (g) nucleus across individual spatial divisions are shown, together with the associated loess-curves (magenta line). The regions displayed in (b) and (c) are highlighted within (a) by the white and orange dashed lines, respectively. Image data have undergone a non-linear transformation to improve visual appearance. Additional file 2: Figure S3. Human epidermis (Patient Two) labeled against phospho-MEK-1/2 (pS218/pS222) using immunofluorescence labeling with confocal microscopy imaging. (a, b) Immunofluorescence images are displayed together with (c) a surface rendering of the signal intensity within suprabasal keratinocytes). The *z*-score normalized sampled signal intensity data (data points; plotted relative to the sample mean, $\overline{\mu}$; and sample standard deviation, $\overline{\sigma}$) and loess smoothed signals (solid lines) associated with the (d) cytoplasm and (f) nuclei are displayed for Patient One (red), Two (green) and Three (blue), together with the 90 % confidence interval for positive and negative residuals (dashed lines), and the sampled data clouds for Patient Two. Histograms of the discretized signal intensity within the (e) cytoplasm and (g) nuclei across individual spatial divisions are shown, together with the associated loess-curves (magenta line). The regions displayed in (b) and (c) are highlighted within (a) by the white and orange dashed lines, respectively. Scale bars represent 10 μm. Image data have undergone a non-linear transformation to improve visual appearance. Basal cells with a relatively-high phospho-MEK-1/2 signal intensity are highlighted (red arrowhead). Additional file 3: Figure S4. Human epidermis (Patient One) labeled against phospho-ERK-1/2 (pT183/pY185) using immunofluorescence labeling with confocal microscopy imaging. (a, b) Immunofluorescence images are displayed together with (c) a surface rendering of the signal intensity within a suprabasal keratinocyte). The *z*-score normalized sampled signal intensity data (data points; plotted relative to the sample mean, $\overline{\mu}$; and sample standard deviation, $\overline{\sigma}$) and loess smoothed signals (solid lines) associated with the (d) cytoplasm and (f) nuclei are displayed for Patient One (red), Two (green) and Three (blue), together with the 90 % confidence interval for positive and negative residuals (dashed lines), and the sampled data clouds for Patient One. Histograms of the discretized signal intensity within the (e) cytoplasm and (g) nuclei across individual spatial divisions are shown, together with the associated loess-curves (magenta line). The regions displayed in (b) and (c) are highlighted within (a) by the white and orange dashed lines, respectively. Scale bars represent 10 μm. Image data have undergone a non-linear transformation to improve visual appearance. Additional file 4: Figure S5. Human epidermis (Patient One) labeled against calmodulin using immunofluorescence labeling with confocal microscopy imaging. (a, b) Immunofluorescence images are displayed together with (c) a surface rendering of the signal intensity and (d) an isosurface volume rendering of a basal keratinocyte. The *z*-score normalized sampled signal intensity data (data points; plotted relative to the sample mean, $\overline{\mu}$; and sample standard deviation, $\overline{\sigma}$) and loess smoothed signals (solid lines) associated with the (e) cytoplasm, (g) nuclei and (i) plasma-membrane are displayed for Patient One (red), Two (green) and Three (blue), together with the 90 % confidence interval for positive and negative residuals (dashed lines), and the sampled data clouds for Patient One. Histograms of the discretized signal intensity within the (f) cytoplasm, (h) nuclei and (j) plasma membrane across individual spatial divisions are shown, together with the associated loess-curves (magenta line). The regions displayed in (b) and (c, d) are highlighted within (a) by the white and orange dashed lines, respectively. Scale bars represent 10 μm. Image data have undergone a non-linear transformation to improve visual appearance. Additional file 5: Figure S6. Absolute thickness of epidermal tissue layers: (a) Basal Layer; (b) Spinous and Granular Layers; (c) Transitional Layer; (d) Total Epidermis. Some variation in the thickness of epidermal tissue layers was observed between patients. To examine this in a quantitative manner, a script was written in MATLAB to move along the lower boundary of each tissue layer and for every unique pixel to measure the minimum distance to the upper boundary (Fig. 2). These results were aggregated over all *z*-positions and target proteins/phospho-proteins, to estimate the distribution of epidermal tissue layer absolute thickness (μm) for Patient One (red), Two (green) and Three (blue). Note that the ‘peakiness’ of these distributions can be attributed to the aggregation of different *z*-positions and target proteins (with minor intra-patient variation for the epidermal thickness between tissue sections). Additional file 6: Table S1. Statistical associations between spatially-conditioned protein abundances used in this study. Statistical associations are italicized if they failed to exceed the data-derived significance thresholds of 0.469 for mutual information, and −0.284 and 0.286 for Pearson’s correlation. As shown in Fig. 3c & d, several relationships between the spatially-conditioned protein abundance data had a statistical association exceeded the data-derived threshold. The canonical interactions between (i) cytoplasmic phospho-Raf and phospho-MEK, and (ii) cytoplasmic phospho-MEK and phospho-ERK had relatively high mutual information and a positive Pearson’s correlation which was particularly strong for (ii). Relationships that reflect nucleocytoplasmic shuttling interactions were also relatively consistent with the known molecular translocation events with ERK-MAPK signal transduction; exceeding the data-derived thresholds for (iii) phospho-ERK-1/2 and (iv) phospho-MEK-1/2, but falling below these thresholds for (v) phospho-Raf-1. Additional file 7: Figure S1. A reaction kinetic scheme of the ERK-MAPK signaling cascade as modeled in this study. The spatial position within the epidermis modulates the relative abundance of Ca^2+^ and plasma-membrane calmodulin. Ca^2+^ activates Ras-GTP signaling, while CaM inhibits signal transduction from Ras-GTP to phosphorylated (pS338) Raf-1 (details given within the main text). The ERK-MAPK signaling cascade with Raf-1 phosphorylating and activating MEK-1/2, which phosphorylates ERK-1/2 is illustrated within the nucleus and cytoplasm; with nucleocytoplasmic shuttling reactions represented by corresponding reactions (dashed lines). Cytoplasmic phospho-ERK-1/2 phosphorylates Raf-1 to inhibit upstream signaling, while nuclear phospho-ERK-1/2 induces the expression of nuclear-localized DUSP4 which promotes ERK-1/2 dephosphorylation. Note that many of the species listed in this model were not measured, and due to the large number of unknown kinetic parameters associated with each reaction (including absolute phospho-protein concentrations), a simplified model was constructed using a normalized Hill differential equation approach (Fig. 1d). Additional file 8: Figure S7. Model simulation in the absence of calmodulin-mediated inhibition of Raf. To test the effects of removing calmodulin-mediated inhibition of phospho-Raf-1, the ‘Hill function weight parameter’ corresponding to this reaction was set to zero using SED-ML, and the model was evaluated at different spatial positions through the epidermis. Note that in comparison to Fig. 4, the relative abundance of phosphorylated ERK-MAPK is increased, and the profile of activation is much more linear, following the Ca^2+^ gradient (at top left). A SED-ML script to perform this simulation is included within the packaged SourceForge code. Additional file 9: Figure S8. Immunofluorescence data derived from whole-cell segmentation. The probability distribution function (p.d.f.) for phospho-MEK signal intensity within the (a) cytoplasm and (b) nucleus of segmented cells. Line color ranges from blue to red, corresponding to the increasing normalized distance value of nucleus centroids. Violin plots are also presented for the phospho-MEK signal intensity within the (c) cytoplasm and (d) nucleus of segmented cells, ordered along the *x*-axis by their normalized distance values (note the blue dashed vertical lines which demarcate the tissue layers, as labeled at bottom of (d), and green dashed lines which highlight ‘phospho-MEK bright basal cells’). Surface renderings of the p.d.f for phospho-MEK signal intensity within the cell (e) cytoplasm and (f) nucleus of segmented cells, plotted perpendicular to the normalized distance values of nuclei centroids (note the plots in surface renderings of the p.d.f are analogous to the ‘whole data clouds segmented by spatial position’, as shown in Additional file 2: Figure S3e and S3g in pMEK.png). Additional file 10: Figure S10. Gradient of keratinocyte cytoplasmic-to-nuclear ratio over the depth of the epidermis. A collection of interfollicular keratinocytes were segmented across multiple *z*-positions within several immunofluorescence data sets, as described in Materials and methods–Estimating cytoplasmic-to-nuclear volume ratio. A linear gradient of increasing cytoplasmic-to-nuclear volume ratio was applied across the depth of the epidermis, increasing from 2 to 5. Note the high degree of variance within the basal layer (Normalized Distance 0–1), which we believe is caused by proliferative cells growing at different stages of the cell cycle. Additional file 11: Figure S9. Human epidermis (Patient One) labeled against MEK-1/2 using immunofluorescence labeling with confocal microscopy imaging. (a, b) Immunofluorescence images are displayed together with (c) a surface rendering of the signal intensity within suprabasal keratinocytes. The *z*-score normalized sampled signal intensity data (data points; plotted relative to the sample mean, $\overline{\mu}$; and sample standard deviation, $\overline{\sigma}$) and loess smoothed signals (solid lines) associated with the (d) cytoplasm and (f) nuclei are displayed for Patient One (red), Two (green) and Three (blue), together with the 90 % confidence interval for positive and negative residuals (dashed lines), and the sampled data clouds for Patient One. Histograms of the discretized signal intensity within the (e) cytoplasm and (g) nuclei across individual spatial divisions are shown, together with the associated loess-curves (magenta line). The regions displayed in (b) and (c) are highlighted within (a) by the white and orange dashed lines, respectively. Scale bars represent 10 μm. Image data have undergone a non-linear transformation to improve visual appearance. NB: the cytoplasmic fluorescence signal intensity (d) shows a strong decrease over the spinous and granular layers, particularly for Patient Two (green) and Three (blue). The inverse pattern for non-phosphorylated MEK-1/2 abundance relative to phospho-MEK-1/2 (Additional file 2: Figure S3d in pMEK.png) indicated that upon pS218/pS222 phosphorylation of MEK-1/2, there may have been masking of the epitope recognized by our total MEK-1/2 antibody. Testing this hypothesis is difficult and beyond the scope of our study, thus we excluded the non-phosphorylated/total ERK-MAPK components from the quantitative modeling.
