# Supplementary figures and images for: Regulation of ERK-MAPK signaling in human epidermis
Source: BMC Syst Biol. 2015 Jul 25;9:41. doi: 10.1186/s12918-015-0187-6 (PMC4514964; doi:10.1186/s12918-015-0187-6)

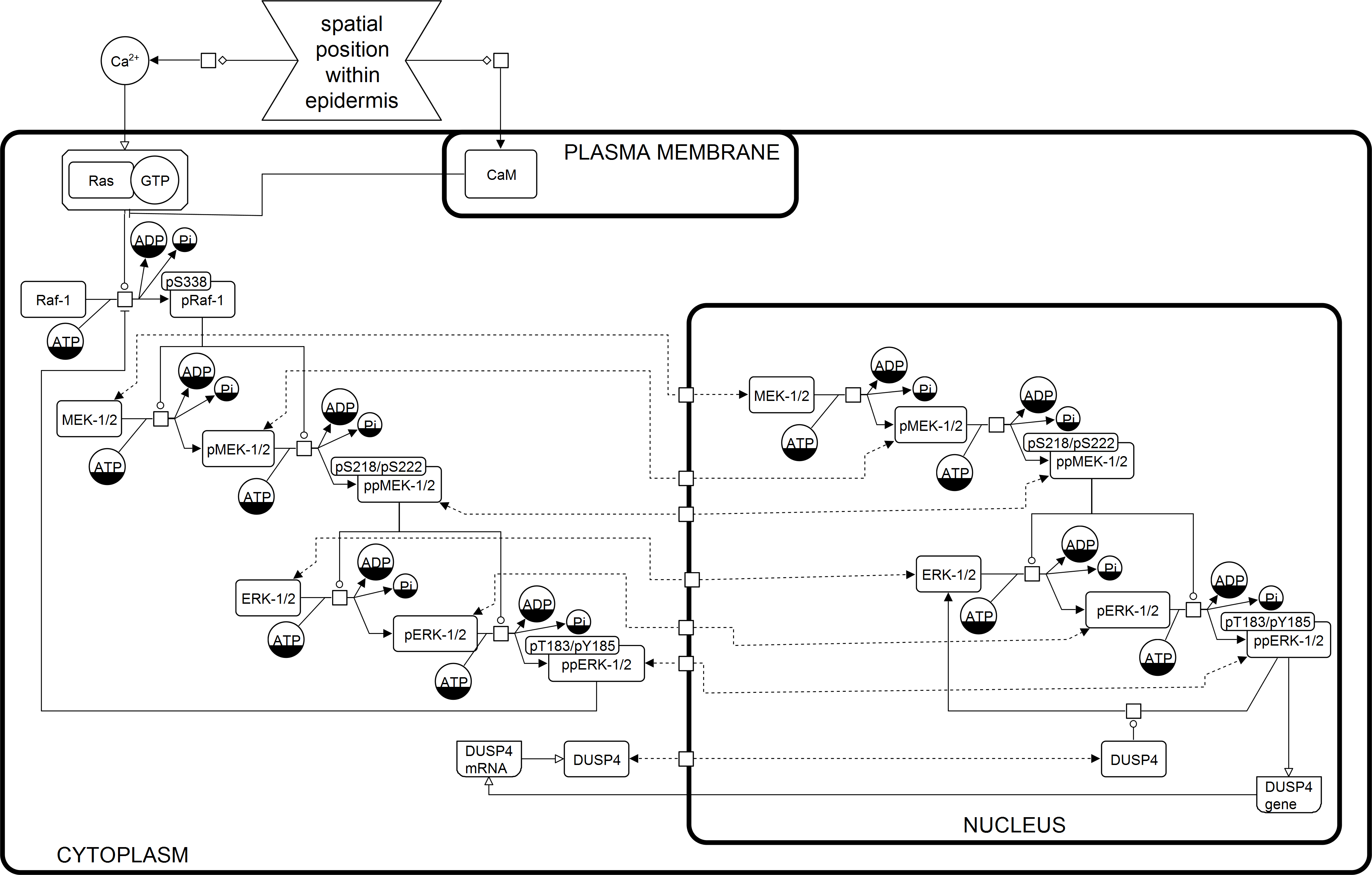

Supplement: Additional file 1: Figure S2. — Human epidermis (Patient Two) labeled against phospho-Raf-1 (pS338) using immunofluorescence labeling with confocal microscopy imaging. (a, b) Immunofluorescence images are displayed together with (c) a surface rendering of the signal intensity within a suprabasal keratinocyte. The z-score normalized sampled signal intensity data (data points; plotted relative to the sample mean, $\overline{\mu}$μ¯; and sample standard deviation, $\overline{\sigma}$σ¯) and loess smoothed signals (solid lines) associated with the (d) cytoplasm and (f) nuclei are displayed for Patient One (red), Two (green) and Three (blue), together with the 90 % confidence interval for positive and negative residuals (dashed lines), and the sampled data clouds for Patient Two. Histograms of the discretized signal intensity within the (e) cytoplasm and (g) nucleus across individual spatial divisions are shown, together with the associated loess-curves (magenta line). The regions displayed in (b) and (c) are highlighted within (a) by the white and orange dashed lines, respectively. Image data have undergone a non-linear transformation to improve visual appearance. [file 12918_2015_187_MOESM1_ESM.png]

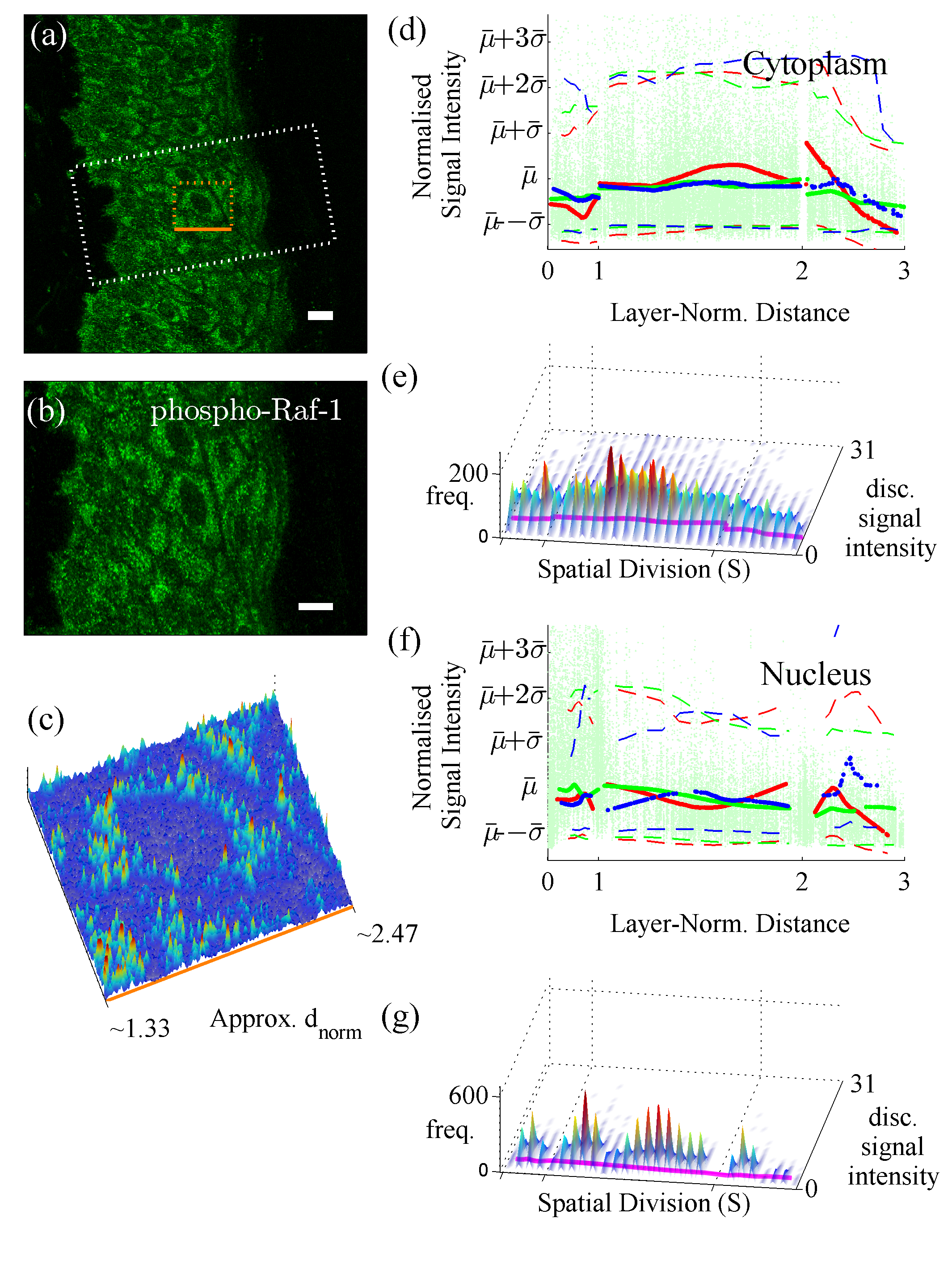

Supplement: Additional file 2: Figure S3. — Human epidermis (Patient Two) labeled against phospho-MEK-1/2 (pS218/pS222) using immunofluorescence labeling with confocal microscopy imaging. (a, b) Immunofluorescence images are displayed together with (c) a surface rendering of the signal intensity within suprabasal keratinocytes). The z-score normalized sampled signal intensity data (data points; plotted relative to the sample mean, $\overline{\mu}$μ¯; and sample standard deviation, $\overline{\sigma}$σ¯) and loess smoothed signals (solid lines) associated with the (d) cytoplasm and (f) nuclei are displayed for Patient One (red), Two (green) and Three (blue), together with the 90 % confidence interval for positive and negative residuals (dashed lines), and the sampled data clouds for Patient Two. Histograms of the discretized signal intensity within the (e) cytoplasm and (g) nuclei across individual spatial divisions are shown, together with the associated loess-curves (magenta line). The regions displayed in (b) and (c) are highlighted within (a) by the white and orange dashed lines, respectively. Scale bars represent 10 μm. Image data have undergone a non-linear transformation to improve visual appearance. Basal cells with a relatively-high phospho-MEK-1/2 signal intensity are highlighted (red arrowhead). [file 12918_2015_187_MOESM2_ESM.png]

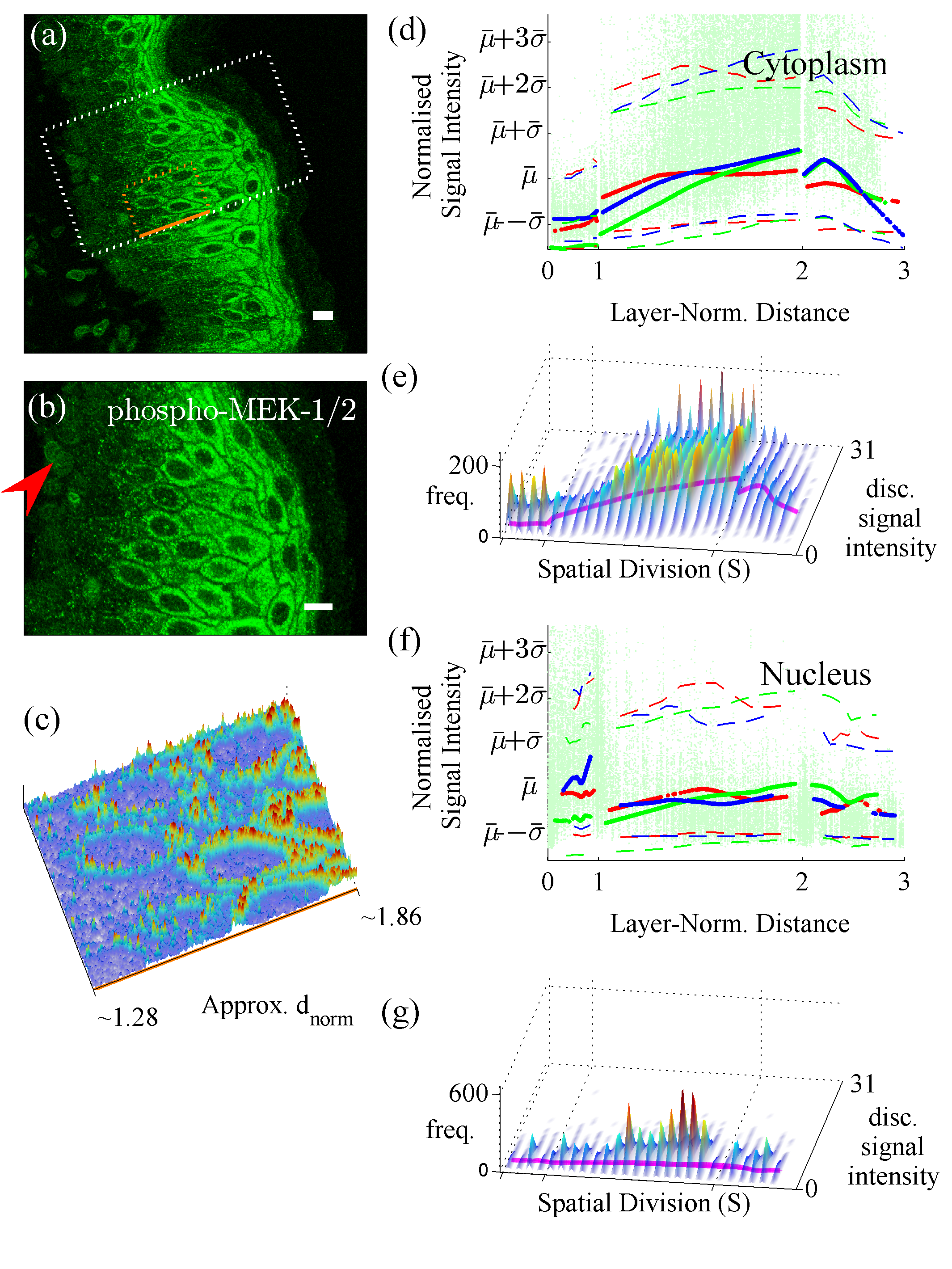

Supplement: Additional file 3: Figure S4. — Human epidermis (Patient One) labeled against phospho-ERK-1/2 (pT183/pY185) using immunofluorescence labeling with confocal microscopy imaging. (a, b) Immunofluorescence images are displayed together with (c) a surface rendering of the signal intensity within a suprabasal keratinocyte). The z-score normalized sampled signal intensity data (data points; plotted relative to the sample mean, $\overline{\mu}$μ¯; and sample standard deviation, $\overline{\sigma}$σ¯) and loess smoothed signals (solid lines) associated with the (d) cytoplasm and (f) nuclei are displayed for Patient One (red), Two (green) and Three (blue), together with the 90 % confidence interval for positive and negative residuals (dashed lines), and the sampled data clouds for Patient One. Histograms of the discretized signal intensity within the (e) cytoplasm and (g) nuclei across individual spatial divisions are shown, together with the associated loess-curves (magenta line). The regions displayed in (b) and (c) are highlighted within (a) by the white and orange dashed lines, respectively. Scale bars represent 10 μm. Image data have undergone a non-linear transformation to improve visual appearance. [file 12918_2015_187_MOESM3_ESM.png]

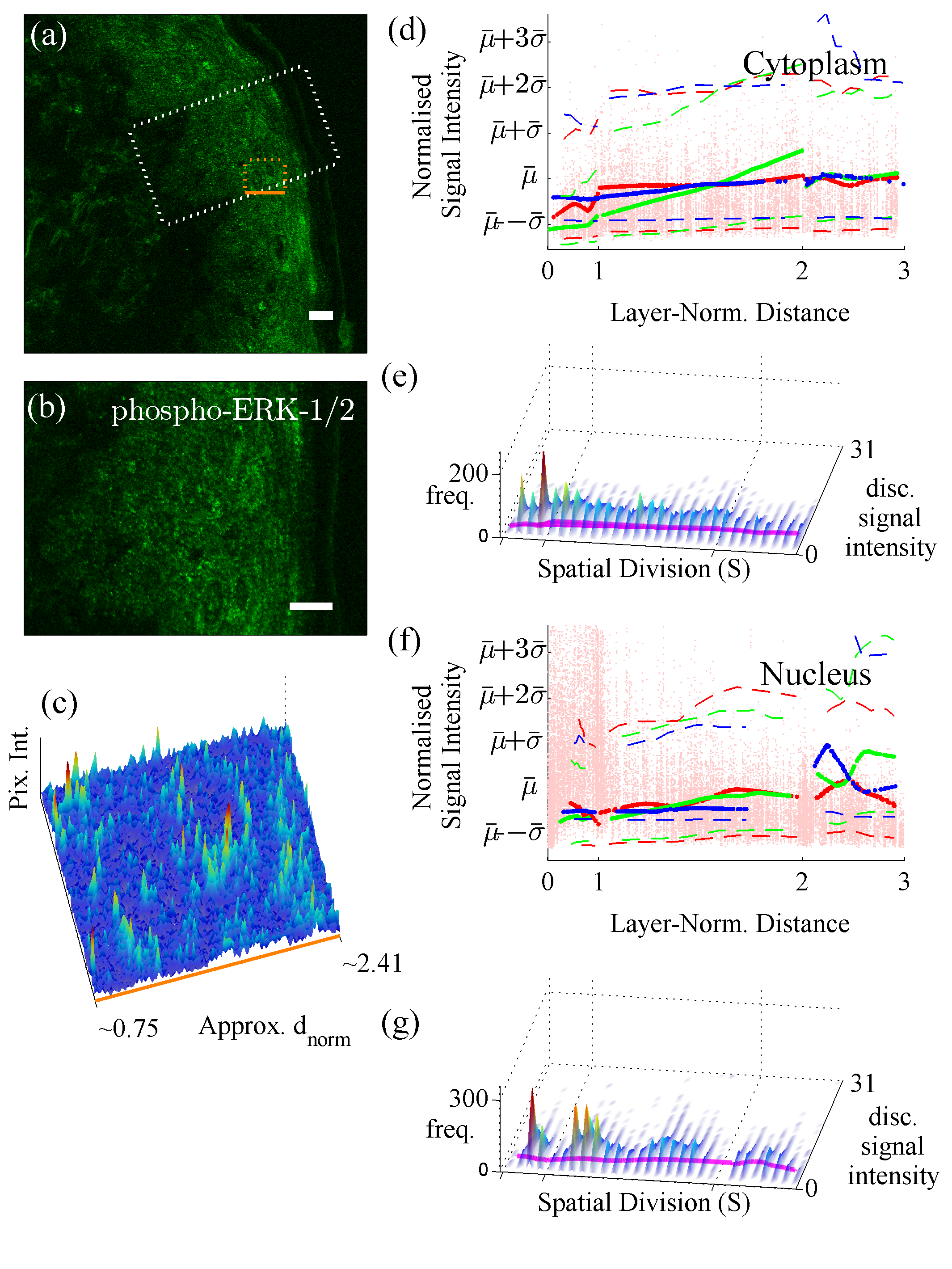

Supplement: Additional file 4: Figure S5. — Human epidermis (Patient One) labeled against calmodulin using immunofluorescence labeling with confocal microscopy imaging. (a, b) Immunofluorescence images are displayed together with (c) a surface rendering of the signal intensity and (d) an isosurface volume rendering of a basal keratinocyte. The z-score normalized sampled signal intensity data (data points; plotted relative to the sample mean, $\overline{\mu}$μ¯; and sample standard deviation, $\overline{\sigma}$σ¯) and loess smoothed signals (solid lines) associated with the (e) cytoplasm, (g) nuclei and (i) plasma-membrane are displayed for Patient One (red), Two (green) and Three (blue), together with the 90 % confidence interval for positive and negative residuals (dashed lines), and the sampled data clouds for Patient One. Histograms of the discretized signal intensity within the (f) cytoplasm, (h) nuclei and (j) plasma membrane across individual spatial divisions are shown, together with the associated loess-curves (magenta line). The regions displayed in (b) and (c, d) are highlighted within (a) by the white and orange dashed lines, respectively. Scale bars represent 10 μm. Image data have undergone a non-linear transformation to improve visual appearance. [file 12918_2015_187_MOESM4_ESM.png]

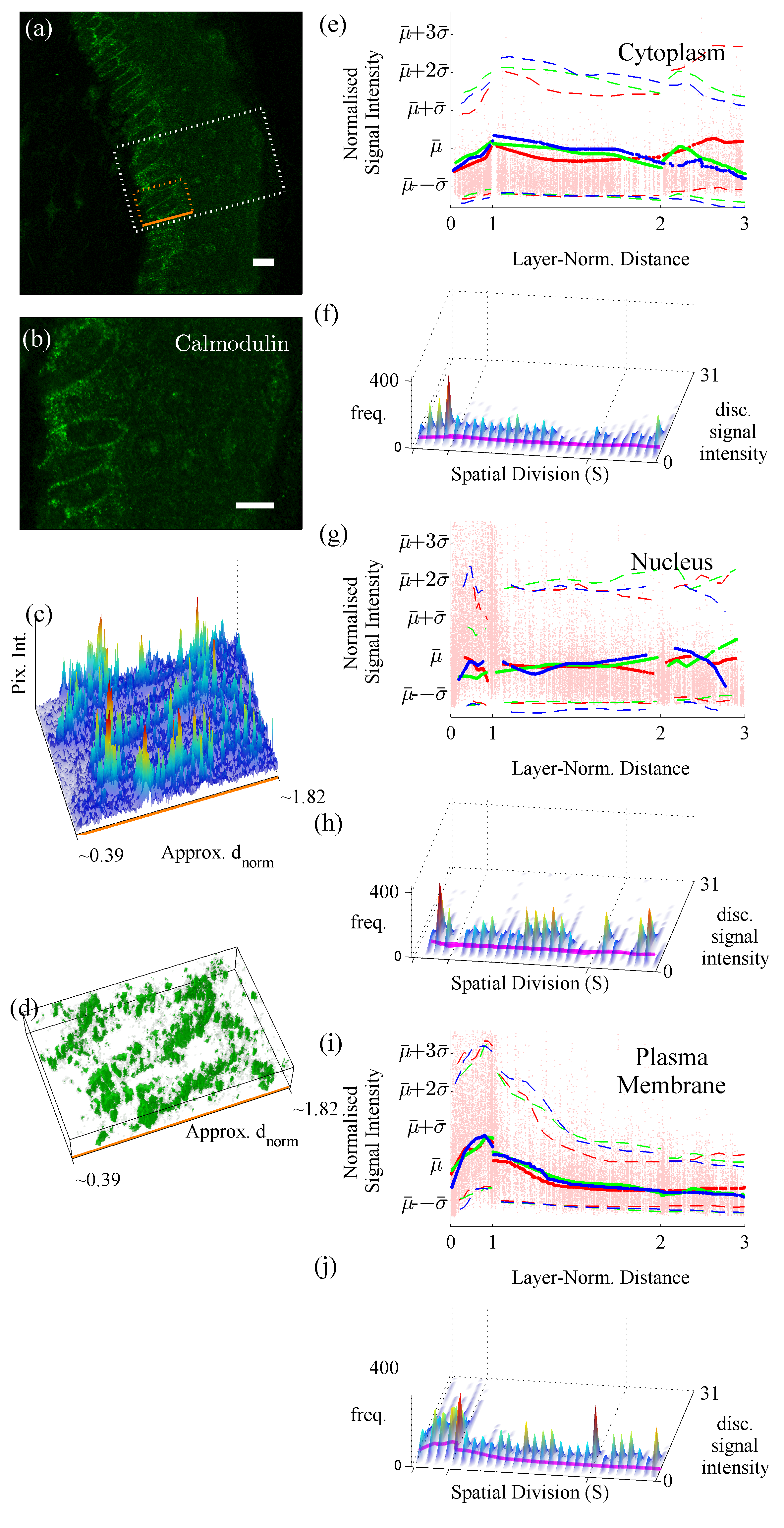

Supplement: Additional file 5: Figure S6. — Absolute thickness of epidermal tissue layers: (a) Basal Layer; (b) Spinous and Granular Layers; (c) Transitional Layer; (d) Total Epidermis. Some variation in the thickness of epidermal tissue layers was observed between patients. To examine this in a quantitative manner, a script was written in MATLAB to move along the lower boundary of each tissue layer and for every unique pixel to measure the minimum distance to the upper boundary (Fig. 2). These results were aggregated over all z-positions and target proteins/phospho-proteins, to estimate the distribution of epidermal tissue layer absolute thickness (μm) for Patient One (red), Two (green) and Three (blue). Note that the ‘peakiness’ of these distributions can be attributed to the aggregation of different z-positions and target proteins (with minor intra-patient variation for the epidermal thickness between tissue sections). [file 12918_2015_187_MOESM5_ESM.png]

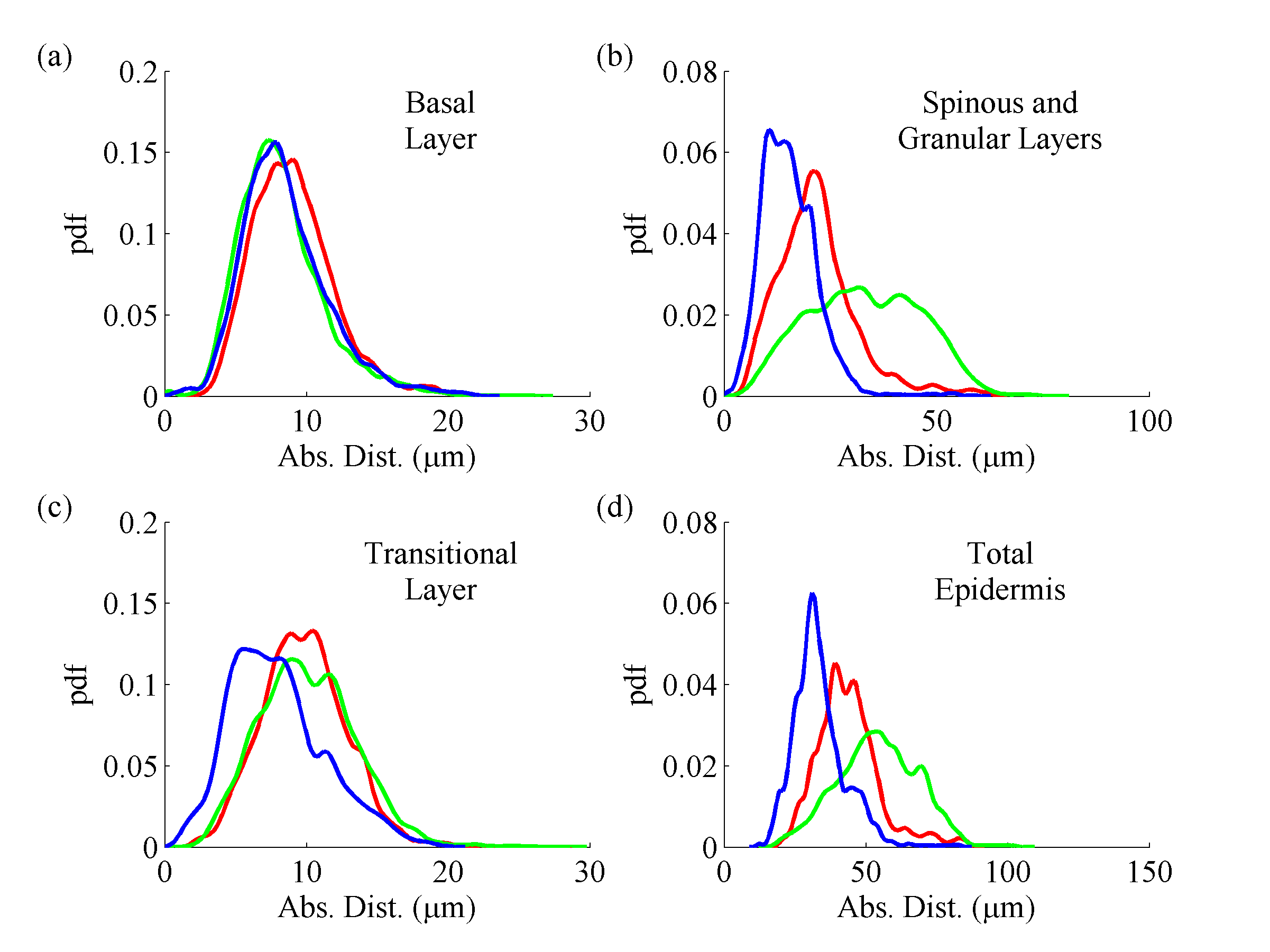

Supplement: Additional file 6: Table S1. — Statistical associations between spatially-conditioned protein abundances used in this study. Statistical associations are italicized if they failed to exceed the data-derived significance thresholds of 0.469 for mutual information, and −0.284 and 0.286 for Pearson’s correlation. As shown in Fig. 3c & d, several relationships between the spatially-conditioned protein abundance data had a statistical association exceeded the data-derived threshold. The canonical interactions between (i) cytoplasmic phospho-Raf and phospho-MEK, and (ii) cytoplasmic phospho-MEK and phospho-ERK had relatively high mutual information and a positive Pearson’s correlation which was particularly strong for (ii). Relationships that reflect nucleocytoplasmic shuttling interactions were also relatively consistent with the known molecular translocation events with ERK-MAPK signal transduction; exceeding the data-derived thresholds for (iii) phospho-ERK-1/2 and (iv) phospho-MEK-1/2, but falling below these thresholds for (v) phospho-Raf-1. [file 12918_2015_187_MOESM6_ESM.docx]

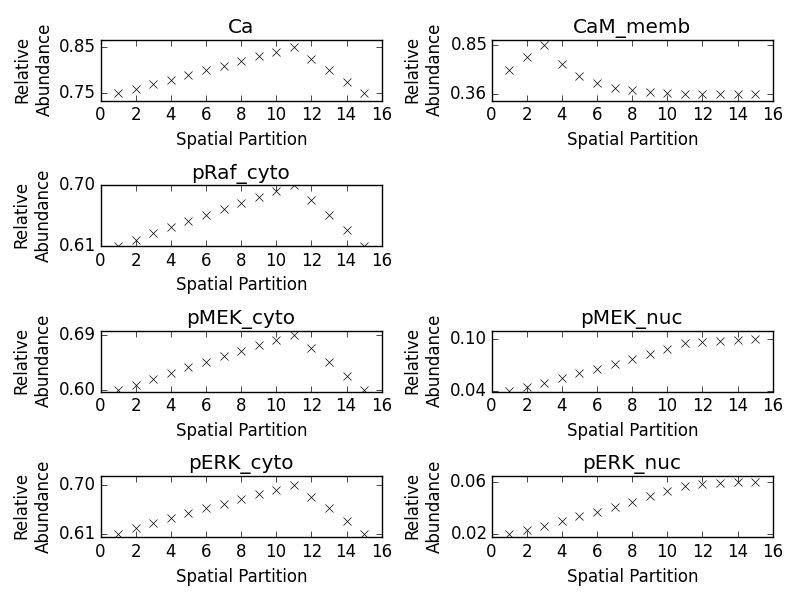

Supplement: Additional file 8: Figure S7. — Model simulation in the absence of calmodulin-mediated inhibition of Raf. To test the effects of removing calmodulin-mediated inhibition of phospho-Raf-1, the ‘Hill function weight parameter’ corresponding to this reaction was set to zero using SED-ML, and the model was evaluated at different spatial positions through the epidermis. Note that in comparison to Fig. 4, the relative abundance of phosphorylated ERK-MAPK is increased, and the profile of activation is much more linear, following the Ca2+ gradient (at top left). A SED-ML script to perform this simulation is included within the packaged SourceForge code. [file 12918_2015_187_MOESM8_ESM.png]

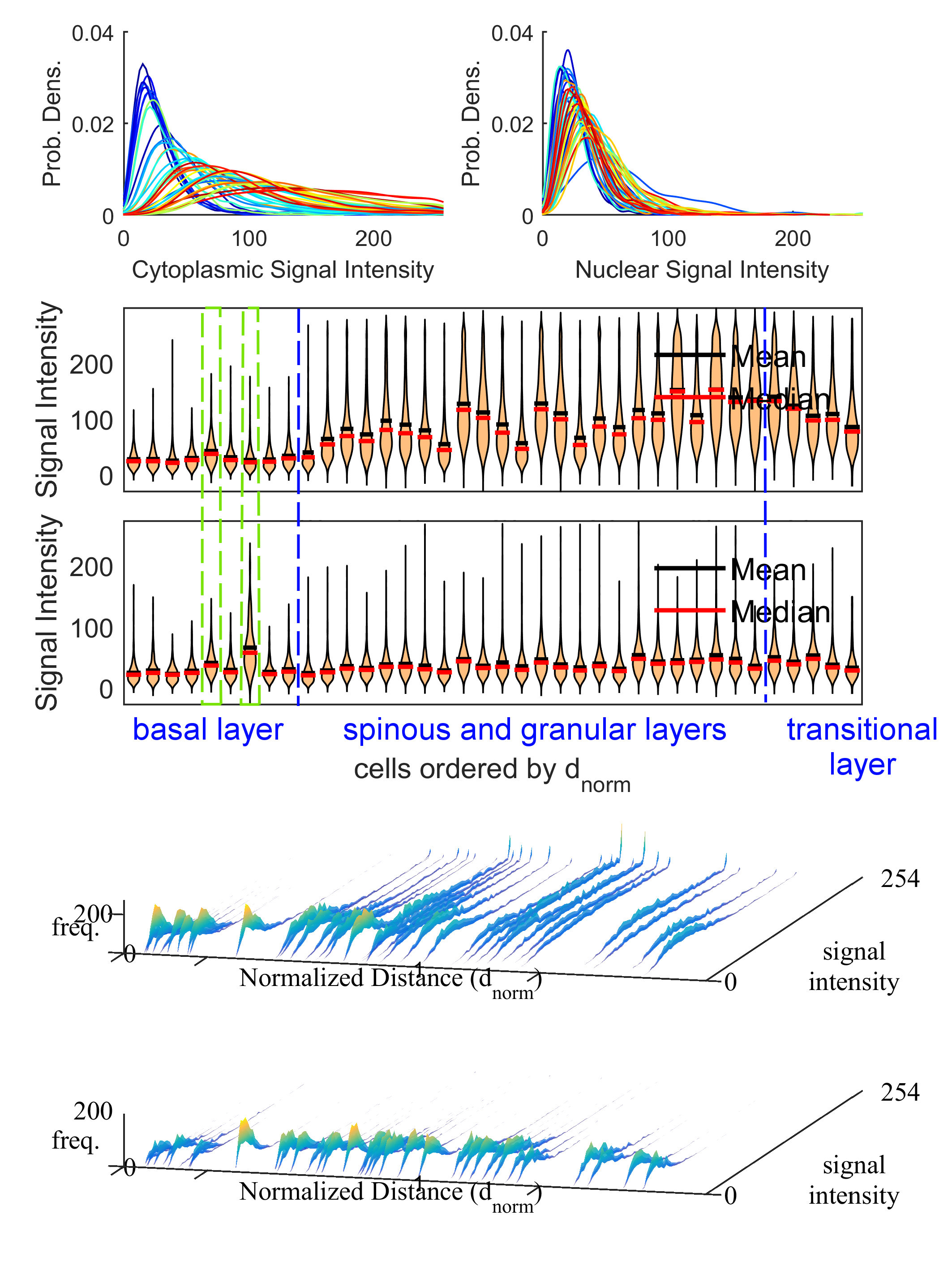

Supplement: Additional file 9: Figure S8. — Immunofluorescence data derived from whole-cell segmentation. The probability distribution function (p.d.f.) for phospho-MEK signal intensity within the (a) cytoplasm and (b) nucleus of segmented cells. Line color ranges from blue to red, corresponding to the increasing normalized distance value of nucleus centroids. Violin plots are also presented for the phospho-MEK signal intensity within the (c) cytoplasm and (d) nucleus of segmented cells, ordered along the x-axis by their normalized distance values (note the blue dashed vertical lines which demarcate the tissue layers, as labeled at bottom of (d), and green dashed lines which highlight ‘phospho-MEK bright basal cells’). Surface renderings of the p.d.f for phospho-MEK signal intensity within the cell (e) cytoplasm and (f) nucleus of segmented cells, plotted perpendicular to the normalized distance values of nuclei centroids (note the plots in surface renderings of the p.d.f are analogous to the ‘whole data clouds segmented by spatial position’, as shown in Additional file 2: Figure S3e and S3g in pMEK.png). [file 12918_2015_187_MOESM9_ESM.png]

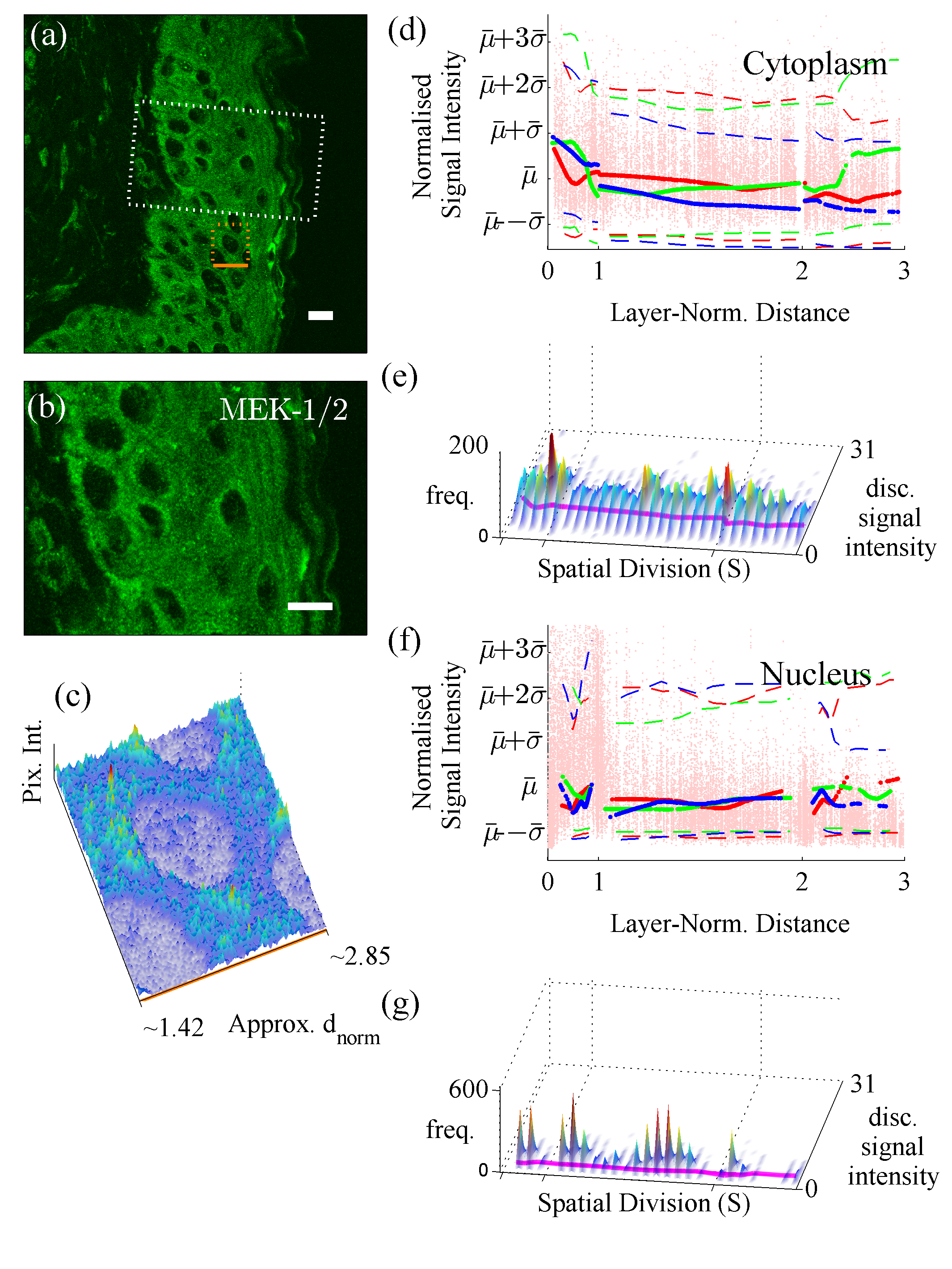

Supplement: Additional file 10: Figure S10. — Gradient of keratinocyte cytoplasmic-to-nuclear ratio over the depth of the epidermis. A collection of interfollicular keratinocytes were segmented across multiple z-positions within several immunofluorescence data sets, as described in Materials and methods–Estimating cytoplasmic-to-nuclear volume ratio. A linear gradient of increasing cytoplasmic-to-nuclear volume ratio was applied across the depth of the epidermis, increasing from 2 to 5. Note the high degree of variance within the basal layer (Normalized Distance 0–1), which we believe is caused by proliferative cells growing at different stages of the cell cycle. [file 12918_2015_187_MOESM10_ESM.png]

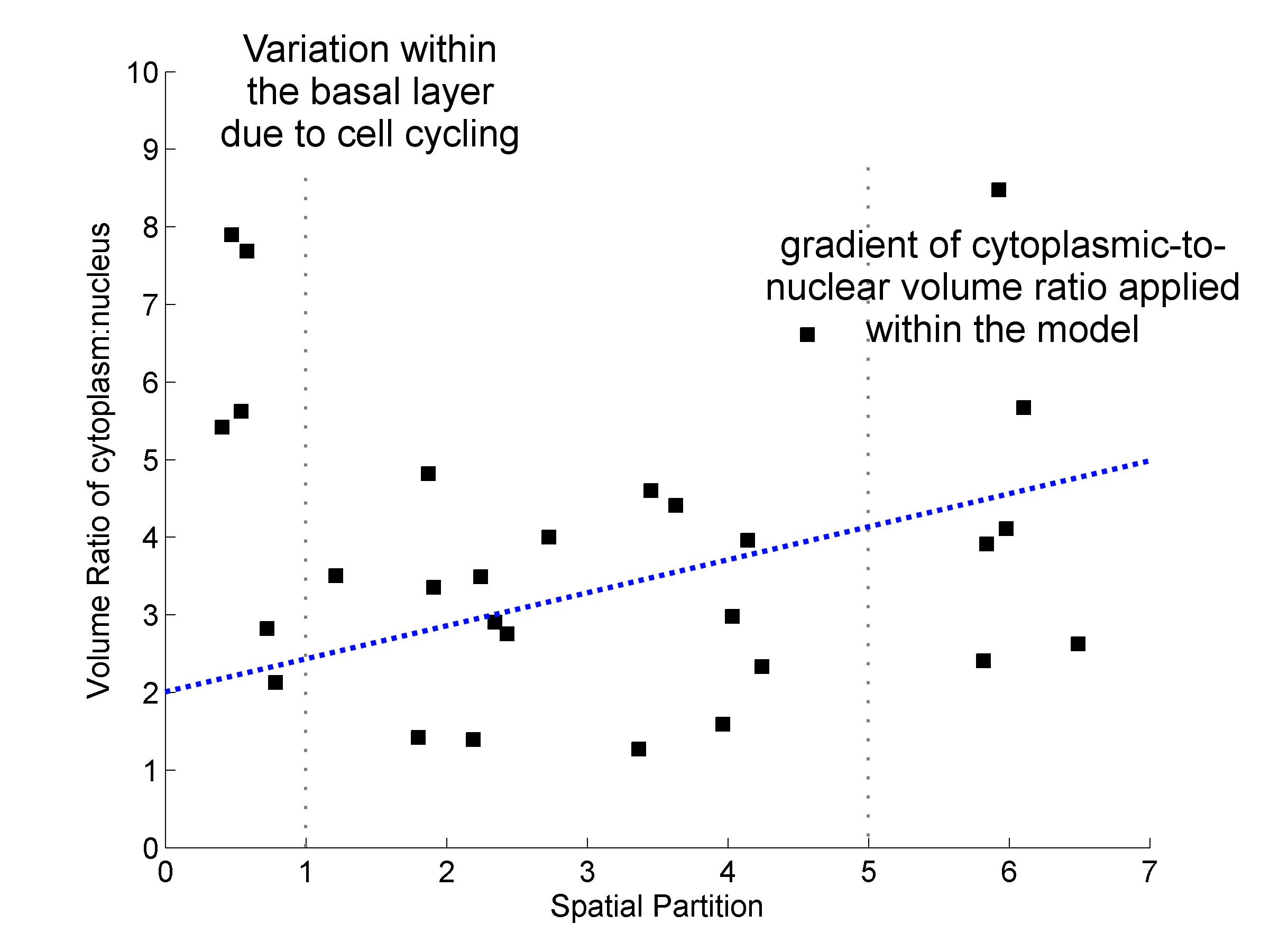

Supplement: Additional file 11: Figure S9. — Human epidermis (Patient One) labeled against MEK-1/2 using immunofluorescence labeling with confocal microscopy imaging. (a, b) Immunofluorescence images are displayed together with (c) a surface rendering of the signal intensity within suprabasal keratinocytes. The z-score normalized sampled signal intensity data (data points; plotted relative to the sample mean, $\overline{\mu}$μ¯; and sample standard deviation, $\overline{\sigma}$σ¯) and loess smoothed signals (solid lines) associated with the (d) cytoplasm and (f) nuclei are displayed for Patient One (red), Two (green) and Three (blue), together with the 90 % confidence interval for positive and negative residuals (dashed lines), and the sampled data clouds for Patient One. Histograms of the discretized signal intensity within the (e) cytoplasm and (g) nuclei across individual spatial divisions are shown, together with the associated loess-curves (magenta line). The regions displayed in (b) and (c) are highlighted within (a) by the white and orange dashed lines, respectively. Scale bars represent 10 μm. Image data have undergone a non-linear transformation to improve visual appearance. NB: the cytoplasmic fluorescence signal intensity (d) shows a strong decrease over the spinous and granular layers, particularly for Patient Two (green) and Three (blue). The inverse pattern for non-phosphorylated MEK-1/2 abundance relative to phospho-MEK-1/2 (Additional file 2: Figure S3d in pMEK.png) indicated that upon pS218/pS222 phosphorylati [file 12918_2015_187_MOESM11_ESM.png]
