# Supplementary material for: Regulation of ERK-MAPK signaling in human epidermis
Source: BMC Syst Biol. 2015 Jul 25;9:41. doi: 10.1186/s12918-015-0187-6 (PMC4514964; doi:10.1186/s12918-015-0187-6)
Supplement: Additional file 7: Figure S1. — A reaction kinetic scheme of the ERK-MAPK signaling cascade as modeled in this study. The spatial position within the epidermis modulates the relative abundance of Ca2+ and plasma-membrane calmodulin. Ca2+ activates Ras-GTP signaling, while CaM inhibits signal transduction from Ras-GTP to phosphorylated (pS338) Raf-1 (details given within the main text). The ERK-MAPK signaling cascade with Raf-1 phosphorylating and activating MEK-1/2, which phosphorylates ERK-1/2 is illustrated within the nucleus and cytoplasm; with nucleocytoplasmic shuttling reactions represented by corresponding reactions (dashed lines). Cytoplasmic phospho-ERK-1/2 phosphorylates Raf-1 to inhibit upstream signaling, while nuclear phospho-ERK-1/2 induces the expression of nuclear-localized DUSP4 which promotes ERK-1/2 dephosphorylation. Note that many of the species listed in this model were not measured, and due to the large number of unknown kinetic parameters associated with each reaction (including absolute phospho-protein concentrations), a simplified model was constructed using a normalized Hill differential equation approach (Fig. 1d). [file 12918_2015_187_MOESM7_ESM.png]

|  | **Association** | **Mutual Information** | **Pearson’s Correlation** |
| --- | --- | --- | --- |
|  | pERK_cyto_ $\leftrightarrow$ CaM_memb_ | 0.90 | -0.79 |
| (*ii*) | pMEK_cyto_ $\leftrightarrow$ pERK_cyto_ | 0.85 | 0.80 |
|  | pERK_nuc_ $\leftrightarrow$ CaM_memb_ | 0.77 | -0.69 |
|  | pMEK_cyto_ $\leftrightarrow$ CaM_memb_ | 0.70 | -0.69 |
| (*i*) | pRaf_cyto_ $\leftrightarrow$ pMEK_cyto_ | 0.67 | 0.46 |
| (*iii*) | pERK_cyto_$\leftrightarrow$ pERK_nuc_ | 0.67 | 0.61 |
|  | pRaf_cyto_ $\leftrightarrow$ pERK_nuc_ | 0.60 | *-0.12* |
|  | pMEK_cyto_ $\leftrightarrow$ pERK_nuc_ | 0.55 | 0.33 |
|  | pRaf_nuc_ $\leftrightarrow$ pMEK_nuc_ | 0.52 | -0.29 |
|  | pMEK_nuc_ $\leftrightarrow$ CaM_memb_ | 0.50 | *-0.06* |
| (*iv*) | pMEK_cyto_ $\leftrightarrow$ pMEK_nuc_ | 0.49 | 0.42 |
|  | pERK_cyto_ $\leftrightarrow$ pMEK_nuc_ | *0.45* | 0.37 |
|  | pRaf_cyto_ $\leftrightarrow$ CaM_memb_ | *0.44* | *-0.15* |
|  | pRaf_nuc_ $\leftrightarrow$ pERK_nuc_ | *0.43* | *-0.19* |
|  | pRaf_cyto_ $\leftrightarrow$ pERK_cyto_ | *0.41* | *0.16* |
|  | pMEK_cyto_ $\leftrightarrow$pRaf_nuc_ | *0.40* | *-0.22* |
|  | pRaf_nuc_ $\leftrightarrow$ CaM_memb_ | *0.39* | *0.22* |
| (*v*) | pRaf_cyto_ $\leftrightarrow$ pRaf_nuc_ | *0.38* | *0.24* |
|  | pRaf_cyto_ $\leftrightarrow$ pMEK_nuc_ | *0.37* | *0.14* |
|  | pMEK_nuc_ $\leftrightarrow$ pERK_nuc_ | *0.35* | *0.17* |
|  | pERK_cyto_ $\leftrightarrow$ pRaf_nuc_ | *0.31* | -0.32 |

**Table S1. Statistical associations between spatially-conditioned protein abundances used in this study.** Statistical associations are italicized if they failed to exceed the data-derived significance thresholds of 0.469 for mutual information, and -0.284 and 0.286 for Pearson’s correlation. As shown in Fig. 3*c* & 3*d*, several relationships between the spatially-conditioned protein abundance data had a statistical association exceeded the data-derived threshold. The canonical interactions between (**i**) cytoplasmic phospho-Raf and phospho-MEK, and (**ii**) cytoplasmic phospho-MEK and phospho-ERK had relatively high mutual information and a positive Pearson’s correlation which was particularly strong for (**ii**). Relationships that reflect nucleocytoplasmic shuttling interactions were also relatively consistent with the known molecular translocation events with ERK-MAPK signal transduction; exceeding the data-derived thresholds for (**iii**) phospho-ERK-1/2 and (**iv**) phospho-MEK-1/2, but falling below these thresholds for (**v**) phospho-Raf-1.
